# Functional studies of plant transcription factors and their relevance in the plant root-knot nematode interaction

**DOI:** 10.3389/fpls.2024.1370532

**Published:** 2024-05-08

**Authors:** Jose Domínguez-Figueroa, Almudena Gómez-Rojas, Carolina Escobar

**Affiliations:** ^1^Facultad de Ciencias Ambientales y Bioquímica, Universidad de Castilla-La Mancha, Toledo, Spain; ^2^Centro de Biotecnologia y Genomica de Plantas (CBGP), Universidad Politecnica de Madrid and Instituto de Investigacion y Tecnologia Agraria y Alimentaria-Consejo Superior de investigaciones Cientificas (UPM-INIA/CSIC), Madrid, Spain

**Keywords:** plant-RKNs interaction, galls, giant cells, transcription factors, new organogenesis, plant defense, plant-development

## Abstract

Root-knot nematodes are polyphagous parasitic nematodes that cause severe losses in the agriculture worldwide. They enter the root in the elongation zone and subtly migrate to the root meristem where they reach the vascular cylinder and establish a feeding site called gall. Inside the galls they induce a group of transfer cells that serve to nurture them along their parasitic stage, the giant cells. Galls and giant cells develop through a process of post-embryogenic organogenesis that involves manipulating different genetic regulatory networks within the cells, some of them through hijacking some molecular transducers of established plant developmental processes, such as lateral root formation or root regeneration. Galls/giant cells formation involves different mechanisms orchestrated by the nematode´s effectors that generate diverse plant responses in different plant tissues, some of them include sophisticated mechanisms to overcome plant defenses. Yet, the plant-nematode interaction is normally accompanied to dramatic transcriptomic changes within the galls and giant cells. It is therefore expected a key regulatory role of plant-transcription factors, coordinating both, the new organogenesis process induced by the RKNs and the plant response against the nematode. Knowing the role of plant-transcription factors participating in this process becomes essential for a clear understanding of the plant-RKNs interaction and provides an opportunity for the future development and design of directed control strategies. In this review, we present the existing knowledge of the TFs with a functional role in the plant-RKN interaction through a comprehensive analysis of current scientific literature and available transcriptomic data.

## Introduction

Plant-parasitic nematodes have the ability to infect a wide range of host plants from which they feed depleting their resources, resulting in significant economic losses in agricultural production worldwide (Singh et al., 2015; Kikuchi et al., 2017). Among these destructive pathogens, the endoparasitic Root-Knot Nematodes (RKNs; *Meloidogyne* spp.) are one of the most economically impactful (Elling, 2013). RKNs, use their stylet and a diverse range of effectors to invade the plant roots and initiate the formation of specialized feeding cells known as giant cells (GCs). These GCs are contained within a novel pseudo-organ called gall that constitutes their feeding site (Escobar et al., 2015). While the significant role of the pericycle in gall formation is well-established from experiments with transgenic lines that induce chemical ablation, the precise origin of the GCs precursor cells remains not fully understood. However, some evidence points to their origin from precursor cells of the pericycle, xylem and/or vascular cambium (Cabrera et al., 2014b; Olmo et al., 2017, Olmo et al., 2020). GCs undergo mitosis accompanied by incomplete cytokinesis and DNA endoreduplication forming a multinucleated cell with greatly increased volume and a dense cytosol. Moreover, GCs also show fragmented vacuoles, undergo cell wall modifications, and ultimately develop membrane invaginations, becoming transfer cells to nourish the nematode (de Almeida Engler and Favery, 2011; Cabrera et al., 2014b; Escobar et al., 2015). RKNs employ sophisticated mechanisms to overcome plant defenses and modulate the host biochemistry and physiology (Kikuchi et al., 2017). They manipulate different genetic regulatory programs of the plant cells, including the cell cycle, various developmental programs, and stress responses, in order to undergo new post-embryogenic organogenesis leading to gall formation. For instance, *Meloidogyne javanica* infection alters pathways involved in *de novo* organogenesis leading to feeding site formation by interfering with auxins signaling cascades (Cabrera et al., 2014b). Consequently, a substantial transcriptional response is triggered (e.g., in *Arabidopsis thaliana*; Jammes et al., 2005; Fuller et al., 2007; Barcala et al., 2010; Silva et al., 2022).

Due to the mentioned dramatic transcriptomic changes described in galls, a pivotal regulatory role of transcription factors (TFs) is therefore expected, coordinating both, the new organogenesis process induced by the RKNs and the plant response against the nematode. Therefore, understanding the role of TFs during RKN infection is essential for a deeper understanding of the plant-nematode interaction and for the development of future control strategies. In this review, we have explored the existing knowledge on TFs involved in the plant-RKN interaction through a comprehensive analysis of current scientific literature and available transcriptomic data, the latter focused on Arabidopsis as considerable transcriptomic data is available and it was shown to be a good model of the plant-RKN interaction (Gheysen and Fenoll, 2011). Our aim is to compile the existing knowledge regarding the crucial role of TFs in the orchestration of the transcriptional response activated within the plant after RKN infection.

## Transcriptional profiling of transcription factors families in arabidopsis

**Figure 1:**
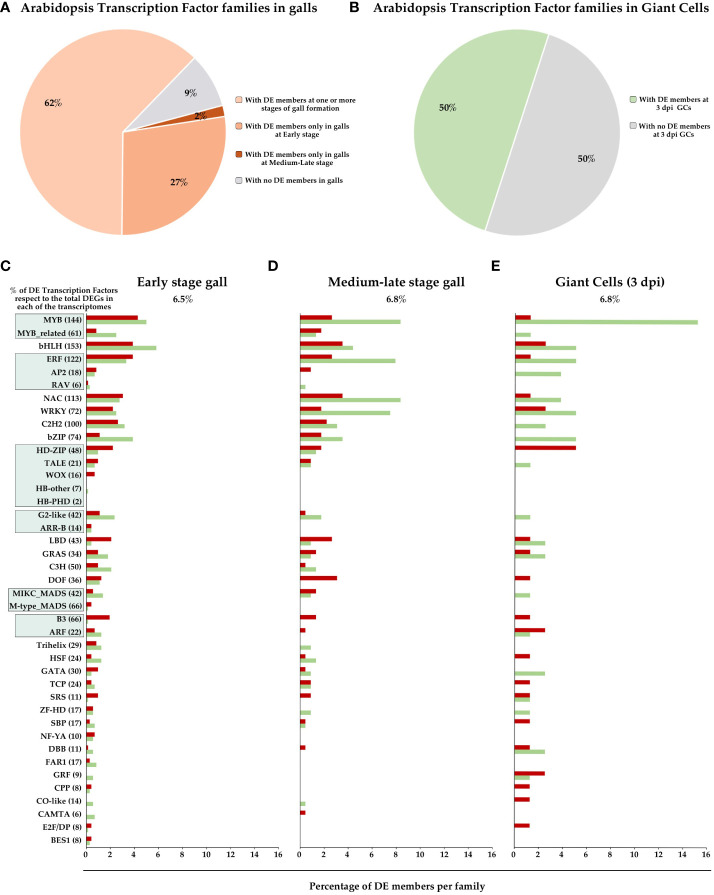
Overview of Arabidopsis Transcription Factor (TF) family profiles in gall formation. Percentage of TF families (58 families according to PlantTFDB; Jin et al., 2014) with and without differentially expressed (DE) members in different gall developmental stages as indicated **(A)** and GCs at 3 dpi **(B)**. The top 36 TF families and the top 6 superfamilies with the highest number of DE members in the RKNs transcriptomes, at early-stage galls [3 dpi galls; **(C)**], galls at mid-late stages [7, 14 and 21 dpi; **(D)**] and GCs 3 dpi **(E)** were represented. X axis, percentage of DE genes in each TFs family in Arabidopsis, with respect to the total members identified in PlantTFDB. Y axis, TFs families and superfamilies indicated by green squares [GARP (G2-like and ARR-B), B3 (ARF and B3), AP2/ERF (RAV, AP2 and ERF), MYB (MYB and MYB_related), MADS (M type, and MIKC) and Homeobox (HD-ZIP, HB-other, TALE, WOX and HB-PHD)] of Arabidopsis according to PlantTFDB. Red, induced genes, green, repressed genes, grey, non-differentially expressed.

Several transcriptomic analyses have been performed to investigate mRNA population changes during RKNs establishment and gall formation in Arabidopsis plants (Jammes et al., 2005; Fuller et al., 2007; Barcala et al., 2010; Silva et al., 2022). These studies have provided valuable insights into the genetic and transcriptional dynamics associated with gall development. Functional classification of differentially expressed genes (DEGs) revealed RNA-related pathways as one of the groups with a high number of DEGs (Barcala et al., 2010), which are mostly involved in biological processes such as transcriptional regulation. Therefore, we analyzed the data contained in the NEMATIC database (NEMatode-Arabidopsis-Transcriptomic-Interaction-Tool; Cabrera et al., 2014a) that includes the most representative transcriptomic experiments of the RKN interaction in Arabidopsis, and the data from a recent RNAseq of galls 3 days post-infection (dpi) in Arabidopsis (Silva et al., 2022). These data show that of the 1717 annotated TF loci in the Arabidopsis genome based on the criteria of the Plant Transcription Factor Data Base (PlantTFDB; Jin et al., 2014), 834 TFs are differentially expressed (DE) in one or more experiments, that correspond to approximately 49% of the total known TFs in Arabidopsis. Among the 58 families classified according to PlantTFDB (Jin et al., 2014), 53 of them have DE members at some stage of gall formation (91%; **Figure 1A**), 52 TFs families at early stage (3 dpi) and 37 at medium-late stages (7, 14 and 21 dpi), (89% and 64%, respectively; **Figure 1A**). In GCs at 3 dpi, 29 TF family members were DE (50%; **Figure 1B**). Only 5 TFs families did not show DE members in either GCs or any of the gall stages. This indicates that most of the TF families are DE at one or more stages of gall and/or GCs formation, which presumably should have a great impact in the dramatic transcriptional changes described in galls (see introduction). **Figures 1C–E** also shows the percentage of DE TFs within the top 36 TF families with the highest number of DE members in early and mid-late stage galls and GCs. Six of these belong to TF superfamilies in which all TF members were included. The predominant families in all three transcriptomes were MYB, bHLH, ERF, NAC and WRKY. The role of several members of these TF families during the plant-nematode interaction was analysed and is our focus throughout the manuscript.

While all these data indicate a substantial involvement of TFs in regulating the transcriptional responses of plants to RKN infection, it is worth noting that the functional roles of only over 30 Arabidopsis TFs and about a dozen in tomato (*Solanum lycopersicum*) have been investigated (**Table 1**). Therefore, our understanding of the regulatory networks orchestrated by TFs in plant-RKN interaction remains rather limited.

**Table 1:** Transcription factors (TFs) analyzed for their functional role in RKNs interaction in different plant species.

TFs family	TF	Plant specie	RKN specie	Promoter activity	Funcional assays	Loss and Gain of function lines	Gall phenotype	Giant cells phenotype	Expression analysis	TFs activity	Reference
WRKY	SlWRKY45	*S. lycopersicum*	*M. javanica*	2, 5, 15, 28 dpi	Yes	Overexpressor lines (*35S:SlWRKY45*)		28 dpi	5, 15, 28 dpi (RNAseq)	Repressor	Chinnapandi et al., 2017
	SlWRKY3			2, 5, 15, 28 dpi	Yes	Overexpressor hairy root lines (*oe:wk-02*; *oe:wk-03*) RNAi silenced lines (*RNAi:wk-03*; *RNAi:wk-04*)			5, 15, 28 dpi (RNAseq)	Activator	Chinnapandi et al., 2019
	SlWRKY35			2, 5, 15, 28 dpi					5, 15, 28 dpi (RNAseq)		
	SlWRKY16	*S. lycopersicum*	*M. javanica*	2, 5, 10, 15, 28 dpi	Yes	Overexpressor hairy root lines (*WRKY16-OE-E2; WRKY16-OE-E5*)			2, 5, and 15 dpi (RNAseq)	Repressor	Kumar et al., 2023
	SlWRKY31					Overexpressor hairy root lines (*WRKY31-OE-E1; WRKY31-OE-E6*)					
	SlWRKY80		*M. incognita*		Yes	VIGS in Motelle and Moneymaker			3, 6 dpi (Motelle versus M82; q-PCR)	Activator	Nie et al., 2023
	SlWRKY72a	*S. lycopersicum*	*Mi-1 virulent M. incognita P77R3*		Yes	VIGS in Motelle and Moneymaker			0, 12, 24, 36 dpi(qRT-PCR)	Activator	Bhattarai et al., 2010
	SlWRKY72b										
	AtWRKY72	*A. thaliana*			Yes	T-DNA insertion lines (*wrky-72-1; wrky72-2*)				Activator	
	SlWRKY70	*S. lycopersicum*	*Mi-1 avirulent M. javanica*		Yes	VIGS in Motelle and Moneymaker			0, 12, 24, 36 hpi (qRT-PCR)	Activator	Atamian et al., 2012
	WRKY11	*A. thaliana*	*M. incognita*	24hpi	Yes	T-DNA insertion lines (*wrky11; wrky11/17*)			24 hpi (qRT-PCR)	Activator	Teixeira et al., 2016
	WRKY17				Yes	T-DNA insertion lines (*wrky11; wrky11/17*)				Activator	
	OsWRKY34	*Oryza sativa*	*M. graminicola*						3, 7 dpi (RNAseq)		Kyndt et al., 2012
	OsWRKY36										
	OsWRKY62										
ERF	ERF109	*A. thaliana*	*M. incognita*	1 dpi, initiation and gall formation	Yes	T-DNA insertion line (*erf109*)				Activator	Zhou et al., 2019; Ribeiro et al., 2024
			*M. incognita*	(3, 5, 7, 10, 14, 21 dpi)							
	ERF115		*M. incognita*	1 dpi, initiation and gall formation	Yes	Dominant repressor line (*35S:ERF115-SRDX*); T-DNA insertion lines (*erf115*, *erf115/ pat1-2*), *ERF115* overexpressing line	7, 14, 21 dpi	30 - 40 dpi		Activator	
			*M. incognita*	(3, 5, 7, 10, 14, 21 dpi)							
	ERF114		*M. incognita*	3, 5, 7, 10, 14, 21 dpi	Yes	*ERF114* overexpressing line	7, 14, 21 dpi	30 - 40 dpi		Activator	Ribeiro et al., 2024
	ERF6		*M. incognita*		Yes	T-DNA insertion line (*erf6-1*)			7 dpi (qRT-PCR) 0,7dpi (Microarray)		Warmerdam et al., 2019
	PUCHI		*RKN*	1, 2, 3, 5, 7 dpi	Yes	T-DNA insertion line (*puchi-1*, TILLING line *puchi-2*)	14 dpi	3, 5, 7, 28-42 dpi	1, 2, 3, 5, 7 dpi	Activator	Suzuki et al., 2021b
MYB	MYB3R1	*A. thaliana*	*M. incognita*		Yes	T-DNA insertion lines (*myb3r1; myb3r1/4; myb3r1/3/5)*					Suzuki et al., 2021a
	MYB3R3			7 dpi	Yes	T-DNA insertion lines (*myb3r3; myb3r3/5; myb3r1/3/5*)				Activator	
	MYB3R4			3, 5, 7 dpi	Yes	T-DNA insertion lines (*myb3r4; myb3r1/4*)				Activator	
	MYB3R5			3, 5, 7 dpi	Yes	T-DNA insertion lines (*myb3r5; myb3r3/5; myb3r1/3/5*)					
	MYB51	*A. thaliana*	*M. incognita*	24 hpi	Yes	T-DNA insertion line (*myb34/51*)			24 hpi (qRT-PCR)	Activator	Teixeira et al., 2016
	MYB34				Yes	T-DNA insertion line (*myb34/51*)			3 dpi (Microarray, RNAseq)	Activator	
ARF	ARF3	*A. thaliana*	*M. javanica*	3 dpi							Cabrera et al., 2016
	ARF5	*A. thaliana*	*M. javanica*	1-14 dpi	Yes	Artificial microRNA line (*ARF5-amiR*), hypomorphic mutated line *arf5-2*, Dominant repressor line (ARF5-SRDX)			7 dpi (qRT-PCR)	Activator	Olmo et al., 2020
												ARF7	1-14 dpi	Yes	Mutagenized seeds lines and T-DNA lines (*arf7-1/arf19-1; nph4-1/arf19-1; slr-1/arf7-1/arf19-1*), gain of function mutation (*slr*)			7 dpi (qRT-PCR)	
	ARF19			1-14 dpi	Yes	Mutagenized seeds lines and T-DNA lines (*arf7-1/arf19-1; nph4-1/arf19-1; slr-1/arf7-1/arf19-1*), gain of function mutation (*slr*)			7 dpi (qRT-PCR)										
												SIARF8A	*S. lycopersicum*	*M. incognita*	7, 14 dpi	Yes	CRISPR lines *(slarf8b, slarf8ab)*		21 dpi	7, 14 dpi (RNAseq)	Activator	Noureddine et al., 2023
	SIARF8B			Yes	CRISPR lines (*slarf8b, slarf8ab*)		21 dpi	7, 14 dpi (RNAseq)	Activator													
	WOX			WOX4	*A. thaliana*	*M. javanica/ M. incognita*	3, 5, 7 dpi	Yes	T-DNA insertion line (*wox4-1*)	7 dpi			7 dpi (RT-PCR)		Yamaguchi et al., 2017
		WOX5	*M. javanica*	2, 5, 8 dpi		Yes	T-DNA insertion line (*wox5-1*)				Activator	Olmo et al., 2020
	GRAS	SCR	*A. thaliana*	*M. javanica*	2, 5, 7 dpi	Yes	*scr-3*				Activator	Olmo et al., 2020
SHR		3,4,7 dpi			Yes	*shr-2*				Activator		
PAT1		*M. incognita*		3, 5, 7, 10, 14, 21 dpi	Yes	*T-DNA insertion lines (pat1-2 and erf115/ pat1-2)*; *PAT1 overexpressing line*	7, 14, 21 dpi	30 - 40 dpi		Activator	Ribeiro et al., 2024
GATA	GATA23	*A. thaliana*	*M. javanica*	1-29 dpi	Yes	RNA intereference line (*GATA23:RNAi*)	15 dpi	15 dpi		Activator	Olmo et al., 2020
G2-LIKE	APL	*A. thaliana*	*M. incognita*	3, 5, 7, 17 dpi						Activator	Suzuki et al., 2021a; Absmanner et al., 2013
HSF	SCZ	*A. thaliana*	*M. javanica*	3, 4, 7dpi 2-40 dpi	Yes	Ac/Ds transposon tagged lines and mutagenized seeds lines (*scz-2; scz1-1; scz-4*)				Activator	Olmo et al., 2020
HD-ZIP	ATHB8	*A. thaliana*	*M. javanica / M. inognita*	3, 5, 7 dpi	Yes	T-DNA insertion line (*athb8-11*)	7 dpi		7 dpi (RT-PCR)		Yamaguchi et al., 2017
DP-E2F-like 1	DEL1	*A. thaliana*	*M. incognita*		Yes	Overexpresor line (*DEL1^OE^*) *del1-1* mutant		7, 14, 21 dpi	7 dpi (*in situ*)	Repressor	de Almeida Engler et al., 2012; Nakagami et al., 2020
												*Prunus Sogdiana*	*M. incognita*						0, 3, 7, 14, 21, 28, 35 dpi (RT-PCR) 0, 3, 7, 14, 21 dpi (*in situ*)		Xiao et al., 2020
		LBD	LBD16	*A. thaliana*	*M. javanica / M. arenaria*	2-29 dpi / 2-45 dpi	Yes	Dominant repressor lines (*35S::LBD16:SDRX; pLBD16:lbd16-SDRX*) T-DNA insertion line (*lbd16-1*)	14 dpi/-	14 dpi/-												Activator	Cabrera et al., 2014b: Olmo et al., 2017
		AP2	TOE1	*A. thaliana*	*M. javanica*		Yes	Overexpression line (*35S::TOE1^R^*)	14 dpi	14 dpi	3 dpi (qPCR)	Repressor	Diaz-Manzano et al., 2018
SBP	SPL7	*A. thaliana*	*M. incognita*	3, 7,14 dpi	Yes	T-DNA insertion line (*spl7*)		7 weeks		Activator	Noureddine et al., 2022

Columns indicate the TF family, the TF name, the plant to which a functional role was assessed, the RKN species used, the infection stages at which promoter activity was confirmed, the stage at which infection and/or reproductive parameters were recorded in lines with altered function, the stage at which gall phenotype was recorded in lines with altered function, the stage at which GCs phenotype was recorded in lines with altered function, the role assigned as activator or repressor, the available expression analysis in infected tissues, and references. An empty cell indicates that no information is available. Dpi, days post infection. hpi, hours post infection. Only in a few cases, no information on TFs functional role was available, however they were included in the table, as they were mentioned within the text.

## Plant transcription factors with a role in plant-defense during the RKNs interaction

Plants have developed a multitude of defense mechanisms to counter potential pathogen attacks. The two primary plant immune responses are PAMP-triggered immunity (PTI), which is initiated by the recognition of receptors that recognize pathogen-associated molecular patterns (PAMPs), and effector-triggered immunity (ETI), in which pathogen effectors are recognized by plant resistance proteins known as R proteins (Peng et al., 2018). Both signaling pathways trigger similar molecular processes such as MAPK cascades, the production of reactive oxygen species, secondary metabolites and an increase in the biosynthesis of hormones such as salicylic acid (SA), jasmonic acid (JA), abscisic acid (ABA) and ethylene (ETH; Peng et al., 2018; Isah, 2019). However, PTI induces rapid and transient activation of MAPKs to enhance local immune responses without triggering plant cell death, whereas ETI results in prolonged and sustained MAPK activity, usually leading to hypersensitive response and programmed cell death (Tsuda et al., 2013). However, both PTI and ETI pathways seem to be interconnected as it has recently been described that the activation of either PTI or ETI alone is not sufficient for effective resistance to the bacterial plant-pathogen *Pseudomonas syringae.* Thus, both immune responses mutually potentiate to activate strong defences against pathogens (Ngou et al., 2020). In any case, plant defense involves a complex interconnected signaling network that ensures a precise transcriptional response. Consequently, several TFs have been identified as crucial for fine-tuning the plant’s transcriptional immune response (Birkenbihl et al., 2017). In this respect, TFs from different families, such as WRKY, MYB, AP2 and bZIP, typically induced in response to various biotic and abiotic stresses (Ambawat et al., 2013; Jiang et al., 2017) were also differentially expressed during the RKNs interaction (e.g., in *Arabidopsis thaliana;* Jammes et al., 2005; Fuller et al., 2007; Silva et al., 2022) including the GCs (Barcala et al., 2010). However, plant-parasitic nematodes, like other pathogens such as bacteria, fungi, oomycetes and some insects, secrete proteins and small molecules, called effectors, to suppress or evade host defense responses and alter host cell structure and function to their advantage, thereby facilitating nematode establishment (Rutter et al., 2022). This section of the review explores the existing literature on defense-related transcription factors (TFs) and their role in the context of the plant-RKNs interaction.

The WRKY family is one of the largest TF families found exclusively in plants. Its members play critical roles in various plant processes, encompassing growth, development, abiotic and biotic stress responses, and plant innate immunity (Wani et al., 2021). Some of them are key components in pathways responsible for PTI and ETI activation (Ribeiro et al., 2022). In an RNAseq data of tomato roots after *M. javanica* infection (15 dpi; Chinnapandi et al., 2017), several WRKYs were identified as negative regulators of the defence response. Among the up-regulated genes, *SlWRKY45* was further studied using a *promoter::GUS* reporter line. The line showed considerable activation at 5 dpi, which continued through feeding-site development and gall maturation (15 and 28 dpi; **Table 1**), in line with the RNAseq data. Two independent overexpressing lines of *35S:SlWRKY45* showed an increase in nematode infection and GCs area compared to the control lines, although the number of GCs within the galls was not affected (Chinnapandi et al., 2017, **Table 1**). Consistently, qRT-PCR revealed a down-regulation of defence-related genes, which are typical markers of JA and SA-mediated pathways, such as those encoding pathogenesis-related (PR-1) and proteinase inhibitor II (Pin2) proteins respectively, which could explain the increased nematode infection in the overexpressing line. In this respect, it has been recently described that SlWRKY45 interact with JA-ZIM domain family proteins that are key repressors of the JA signalling, and it is also able to bind and inhibit the activity of the promoter of the JA biosynthesis gene ALLENE OXIDE CYCLASE (AOC) (Huang et al., 2022). All of this is consistent with the attenuated resistance to *Meloidogyne incognita* of *SlWRKY45* overexpression and confirm its role as a negative regulator fo the defense response (PTI) against *Meloidogyne* spp. Additionally, *SlWRKY45* is upregulated by cytokinins, and its overexpression caused the repression of the cytokinin response factor 1 (*CRF1*) and *CRF6* (Chinnapandi et al., 2017). RKNs and cyst nematodes (CNs) have the ability to synthesize and secrete cytokinins, as noted by De Meutter et al. (2003). Furthermore, it has been demonstrated that the CN *Heterodera shachtii* has a functional cytokinin-synthesizing isopentenyltransferase gene which is essential for virulence and feeding site expansion (Siddique et al., 2015).The secretion of nematode cytokinins could potentially disrupt the balance of plant hormones and cytokinin signalling. Therefore, SlWRK45 may play a crucial role in coordinating hormone signals that promote nematode development within the root tissue. Similarly, recent studies have identified SlWRKY16 and SlWRKY31 as negative regulators of plant immunity and defence (PTI) in tomato. Both genes were induced during nematode infection until late infection stages (28 dpi). Overexpression of these genes in tomato lines using *R. rhizogenes*-mediated transformation under the control of the *CaMV35S* promoter resulted in increased susceptibility to *M. javanica*, as evidenced by enhanced galling and reproduction parameters (Kumar et al., 2023; **Table 1**).

**Figure 2:**
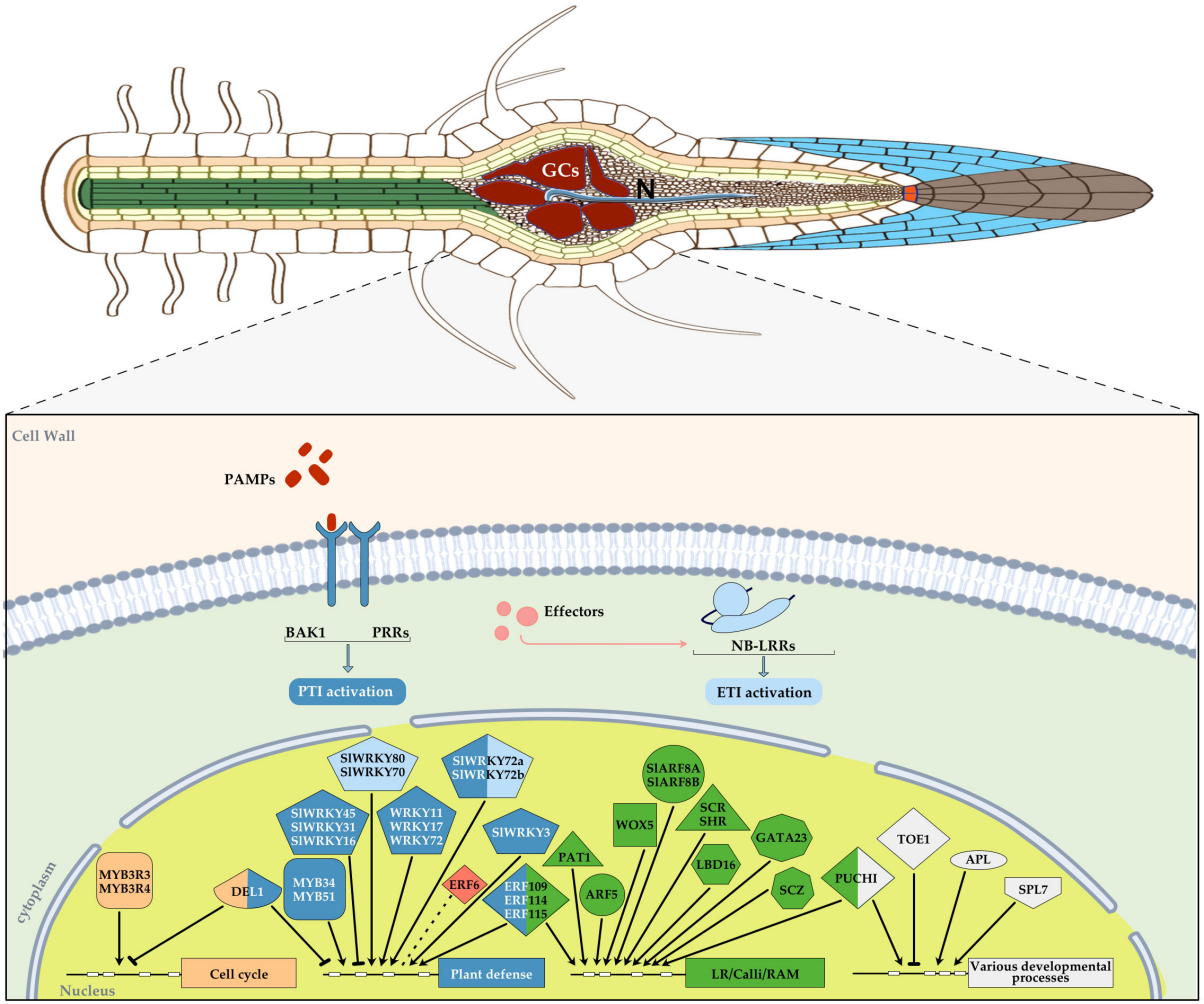
Diagram showing an overview of those transcription factors (TFs) with an assigned function during plant-RKNs interaction in different plant species, mainly *Arabidopsis thaliana* and *Solanum licopersicum*, integrated in a basic diagram of a plant cell. TFs were categorized into different groups according to their function and a color was assigned in each geometric figure, i.e. cell cycle, light orange, plant defense, blue, lateral root/calli/Root apical meristem (RAM), green, various developmental processes, white, abiotic stress, red. In some cases, a dual function was demonstrated and therefore, two colors were assigned. A blunt end arrow indicates a TF with a repressor role and an arrowhead indicates its function as an activator. RAM, root apical meristem, each TF family was assigned a different geometric figure. ETI, light blue, PTI, dark blue.

In contrast to SlWRKY45, 16, and 31, which act as negative regulators of the plant defence response to RKNs, other members of the WRKY family have been described as positive regulators of defence against RKNs. For instance, *WRKY11*, whose expression is induced 24 hours after infection with *M. incognita*, and WRKY17, which can function in partial redundancy with WRKY11, are associated with the activation of basal defence mechanisms (PTI). The Arabidopsis lines *wrky11*, *wrky17*, and *wrky11/wrky17* exhibited increased susceptibility to *M. incognita*. This was evident from the significantly higher number of galls observed 4 weeks after inoculation in both the single and double mutant lines compared to the wild-type plants. However, there were no significant differences between the single mutants and the double mutant, indicating that these two TFs do not function redundantly in this pathogenic interaction (Teixeira et al., 2016; **Table 1**). Similarly, mutant lines *wrky11* and *wrky17* showed more susceptibility in Arabidopsis infected with the CN *Heterodera schachtii* (Ali et al., 2014), indicating commonalities in the basal resistance mechanisms between both plant-(RKNs and CNs) interactions. Additionally, *WRKY11pro::GUS* lines showed that the *WRKY11* promoter was activated in the root elongation zone and root tip 24 hai with *M. incognita* (**Table 1**). Furthermore, in assays based on treatments with crude extracts of J2 larvae, GUS activity was also detected in roots, mainly in the elongation zone where RKNs invade roots. The promoter activity was detected in the absence of any mechanical damage produced by RKNs penetration and intercellular migration (Teixeira et al., 2016). This suggests that the gene is an early responder to the presence of the nematode. Similarly, GUS staining was observed restricted to the root elongation zone of *MYB51pro::GUS* plants early after infection with *M. incognita* (**Table 1**; **Figure 2**). MYB51 is a member of the MYB Transcription Factor family and, together with MYB34, regulates glucosinolate biosynthesis. Accordingly, the Arabidopsis *myb51 myb34* double mutant, which is completely impaired in glucosinolate production (Frerigmann and Gigolashvili, 2014), displayed a higher number of galls compared to wild-type plants in *M. incognita* infection assays. This increased susceptibility of the double mutant point to a positive role of glucosinolates in RKNs defense (Teixeira et al., 2016; **Table 1**; **Figure 2**). Importantly, WRKY11 and MYB51 are involved in BRASSINOSTEROID INSENSITIVE-ASSOCIATED KINASE 1 (BAK1)-dependent PTI responses activated by RKN infection, however, another BAK-independent immune signaling pathway was detected probably involved in the camalexin biosynthetic pathway (Teixeira et al., 2016). It is well-established that nematodes can suppress defense-related genes in feeding cells. In this respect, transcriptomic data of Arabidopsis and tomato micro-dissected GCs induced by *M. javanica* at early infection stages (3 dpi) showed a down-regulation of *MYB34* in Arabidopsis and its ortholog in tomato, as reported by Barcala et al. (2010) and Portillo et al. (2013), respectively. In contrast, the RNAseq analysis conducted by Silva et al. (2022) at the same infection stage, showed that *MYB34* is induced in whole galls compared to control non-infected root segments. These findings suggest that RKNs may inhibit glucosinolate production in feeding cells, while a defense response is likely maintained in the remaining gall tissues. Other WRKY members related to the basal defense against RKNs as positive regulators are *AtWRKY72* and its orthologs in *Solanum lycopersicum*, *SlWRKY72a* and *SlWRKY72b* (Bhattarai et al., 2010; **Table 1**; **Figure 2**). Arabidopsis WRKY72 mutant lines showed a significant increase in egg masses compared to Col-0. Tomato roots of the susceptible cultivar (cv) Moneymaker (*mi-1/mi-1*), with *SlWRKY72a* and/or *SlWRKY72b* transient silenced or co-silenced showed similar results, confirming the involvement of *SlWRKY72* in basal defense (PTI) in both plant species (Bhattarai et al., 2010; **Table 1**). In addition, data obtained with the tomato resistant cv. Motelle (*Mi-1/Mi-1*) indicated that *SlWRKY72* is also involved in gene-for-gene resistance (ETI) during RKN infection of tomato roots. Thus, a significant increase of *SlWRKY72a* expression level was observed in response to *M. incognita* 12 and 36 hours after inoculation (hai), as well as of *SlWRKY72b* expression at 12 hai in tomato roots of the resistant cv. Motelle (*Mi-1/Mi-1*), whereas this was not observed in the susceptible cv. Moneymaker (*mi-1/mi-1*, Bhattarai et al., 2010). Functional confirmation of the participation of both TFs in tomato gene-for-gene resistance was obtained by transient silencing or co-silencing *SlWRKY72a* and *SlWRKY72b* in the resistant Motelle roots. This resulted in an increased susceptibility to RKNs, while no infection was observed in the control Motelle roots. The expression changes of another WRKY family member in tomato, *SlWRKY70*, also suggest a putative role in *Mi-1*-mediated resistance (ETI), as its mRNA was up-regulated in both susceptible and non-susceptible lines and RKNs were able to infect and reproduce in *WRKY70* transiently silenced tomato roots of the resistant cv. Motelle, whereas no infection and reproduction was observed in non-silenced Motelle tomato roots (Atamian et al., 2012; **Table 1**; **Figure 2**). In addition, WRKY70 has been described in Arabidopsis to mediate basal and R gene defence in response to aphids (Knoth et al., 2007). Therefore, both WRKY70 and WRKY72 showed a conserved role as activators of defense in different plant species in response to different pests and pathogens (Knoth et al., 2007; Bhattarai et al., 2010; Atamian et al., 2012). However, they appear to mediate defense through different signaling pathways; in Arabidopsis, WRKY70 acts as a mediator between the SA and JA defense pathways during biotic stress (Li et al., 2006); a similar scenario might be happening in tomato as *SlWRKY70* is up-regulated during the first hours after SA treatment, but repressed after Methyl jasmonate (MeJA) application (Atamian et al., 2012; **Table 1**). On the other hand, WRKY72 seems to be involved in plant defence against pathogens in a SA independent manner as genes deregulated in *Atwrky72* did not respond to SA analogues (Bhattarai et al., 2010).

During the interaction with RKN, other members of the WRKY transcription factor family in tomato, such as *WRKY3* and *35*, were induced and functional, i.e., *SlWRKY3pro::GUS* and *SlWRKY35pro::GUS* lines showed GUS signal at early infection stages with *M. javanica* (2 and 5 dpi). Furthermore, the overexpression of *SlWRKY3* in transgenic hairy root lines led to a decrease in the reproduction of *M. javanica*. This was accompanied by an increase in the accumulation of defence molecules from the shikimate and oxylipin pathways. On the other hand, *SlWRKY3 RNAi* lines promoted reproduction compared to wild-type plants, confirming its role in basal defence (PTI) against RKNs (Chinnapandi et al., 2019; **Table 1**; **Figure 2**). Moreover, SlWRKY80 was also recently identified as a positive regulator that affects *Mi-1*-mediated resistance (ETI). In Motelle lines carrying the *Mi-1* resistance gene, *SlWRKY80* transcript levels increased by more than 3.21 and 4.56-fold at 3 and 6 dpi, respectively, compared to the control reference M82 variety. The expression of *SlWRKY80* was also significantly induced by the defence hormones JA and SA in tomato Motelle. Furthermore, virus-induced gene silencing (VIGS) assays demonstrated that silencing *SlWRKY80* in the resistant Motelle tomato resulted in a significant increase in the number of egg masses in the roots of individual plants and a significant decrease in its resistance level. This confirms the role of SlWRKY80 as a positive regulator of *Mi-1*-mediated tomato resistance (Nie et al., 2023; **Table 1**; **Figure 2**).

Finally, it is possible that WRKY family members are involved in defence responses induced during the infection by RKNs in monocotyledonous species. This is supported by a transcriptomic study of galls at 3 and 7 dpi of *Oryza sativa* infected with *M. graminicola* that revealed upregulation of *OsWRKY34*, *OsWRKY36* and *OsWRKY62* (Kyndt et al., 2012; **Table 1**; **Figure 2**). Yet, further analysis is required to determine the functional implications of these TFs in rice responses against RKNs.

## Plant transcription factors with a dual link to stress caused by RKNs and plant developmental programs

Although, the structure of this review makes a sharp separation between those TFs involved in plant defense and those involved in plant development, the boundaries are not always clear. In recent years, a connection between stress signaling and developmental programs, such as root regeneration, has been extensively described (Ikeuchi et al., 2019). One example of the RKNs interaction involves the ERF109 and ERF115 transcription factors, which belong to the Ethylene Responsive Factor (ERF) subfamily within the APETALA2/ETHYLENE RESPONSIVE FACTOR (AP2/ERF) superfamily (Sakuma et al., 2002; Wu et al., 2022). These transcription factors are involved in a core molecular network triggered by wound-induced JA, which induces stem cell activation and regeneration of *Arabidopsis thaliana* roots (Zhou et al., 2019). ERF109 and ERF115 play a crucial role in maintaining the quiescence of the root stem organizer cells, also known as the quiescent center (QC). ERF109 is activated transcriptionally within minutes in response to JA and wounding and operates upstream of ERF115. Conversely, ERF115 operates upstream of the protein complex RBR-SCR, which regulates the asymmetric cell divisions of root stem cells, QC quiescence, and the activation of the QC regulatory protein WOX5. In addition, it is worth noting that ERF115 is activated not only by JA but also by auxin signaling, which is crucial in galls (Cabrera et al., 2014b; Olmo et al., 2020). It is interesting to observe that GUS staining assays revealed the induction of both ERF109 and ERF115 promoters after the infection of the *M. incognita* as early as 1 dpi. Furthermore, time course experiments have shown that *ERF109* is induced during nematode penetration, while *ERF115* is strongly induced in vascular and/or endodermal cells at all stages from penetration and feeding site initiation until gall formation. Interestingly the repressor activity of ERF115 in the dominant-negative *ERF115-SRDX* line resulted in a loss of root growth recovery capacity after infection with *M. incognita* compared to Col-0. Furthermore, the *erf109* mutant and *ERF115-SRDX* line exhibited reduced susceptibility to infection and developed fewer egg masses compared to Col-0 seedlings 7 weeks after inoculation. Consistently, gall formation in *ERF115-SRDX* roots progressed slower than in Col-0, and DNA synthesis at feeding sites was less active compared to Col-0 (Zhou et al., 2019). Therefore, the JA-induced regeneration pathway, with ERF109 and ERF115 acting as regulators, stimulates root growth following nematode infection and contributes to the reproductive success of *M. incognita* (Zhou et al., 2019; **Table 1**; **Figure 2**). In this respect, it is relevant to mention that the damage caused by *H. schachtii* during invasion also activates a jasmonate-dependent ERF109 pathway, promoting secondary root formation (Guarneri et al., 2022). Therefore, new root-growth and/or regeneration in both RKNs and CNs interaction seems to be mediated by common JA responsive transducers as ERF109. Furthermore, ERF115 interacts with PAT1, a transcription factor belonging to the PHYTOCHROME A TRANSDUCTION 1 (PAT1) GRAS subfamily, forming heterodimers, and *erf115*, *pat1-2*, and *erf115/pat1-2* double mutants showed GCs often with less cytoplasm, fewer nuclei, and less ploidy than the wild-type lines, which probably caused a delay in nematode development. Furthermore, overexpression of *ERF115* (*ERF115OE*), *ERF114*, and *PAT1* resulted in accelerated gall induction and downstream activation of key players in the regenerative pathway, possibly related to the high cell division rates observed, particularly in the *ERF115OE* line, following mechanical stress induced by RKNs. In conclusion, the ERF115-PAT1 complex contributes to the regenerative potential of nematode-induced galls by facilitating tissue healing, thereby maintaining the gall’s functionality until maturation and nematode reproduction (Ribeiro et al., 2024; **Table 1**; **Figure 2**).

*ERF6* is another stress-related gene that does not appear to regulate plant defenses. It is a member of the Ethylene Responsive Factors (ERF) family and has a unique AP2/ERF domain (Nakano et al., 2006). It was identified from a quantitative trait loci (QTL) study that investigated the relationship between allelic variation in specific loci (QTLs) and the susceptibility of Arabidopsis to *M. incognita* (Warmerdam et al., 2019). qPCR analysis showed a significant down-regulation of *ERF6* in Arabidopsis wild type seedling roots at 7 dpi in association with nematode infections. Reproduction tests were conducted on the *erf6-1* mutant line infected with *M. incognita* at 7 dpi, which resulted in a significant increase of 28% in egg masses per plant compared to the wild type line. In addition, microarray analysis revealed that there were 489 differentially expressed genes in the roots of *erf6-1* nematode-infected plants compared to infected wild-type plants (Warmerdam et al., 2019). Previous studies have shown that ERF6 is phosphorylated by MPK3/MPK6 during biotic stress, which activates the expression of Jas/ETH defense genes such as *PDF1.2a* and *PDF1.2b*, thereby enhancing Arabidopsis’ defense against fungal infections (Meng et al., 2013; Wang et al., 2022). However, in *erf6* RKNs-infected roots, the expression of *PDF1.1* and *PDF1.2* was not significantly altered compared to wild-type infected plants. This is similar to the expression of other pathogenesis-related genes, such as *PR1*, *PR2*, *PR3*, and *PR4*, which are known to be regulated by ERFs. These findings suggest that ERF6 does not suppress plant defenses during the RKNs interaction. Many of the genes that are differentially regulated in the roots of nematode-infected *erf6-1* plants at 7 dpi are putatively involved in responses to abiotic stresses, such as osmotic stress. This suggests that ERF6 regulates abiotic stress responses in the plant-nematode interaction (Warmerdam et al., 2019; **Table 1**; **Figure 2**).

## Plant transcription factors relevant during RKNs infection with impact in plant-development

The root-knot nematodes induce feeding cells, GCs, with enlarged nuclei within their heterogeneous feeding sites or feeding organs called galls, which indicates increased DNA replication cycles (de Almeida Engler et al., 1999). This process is called endoreduplication and occurs when successive phases of DNA synthesis (S) follow each other without intervening mitosis or cytokinesis. Endopolyploidy is observed in differentiated and enlarged plant cells, such as Arabidopsis trichomes, endosperm, and fruit (Sabelli et al., 2007). Somatic polyploidy is particularly prevalent in higher plants. A high-resolution DNA endoploidy map of the developing Arabidopsis root has revealed the importance of endoreduplication for the expression of genes encoding cell-wall modifying enzymes. These enzymes are crucial during GC development, suggesting that these responses may serve as a buffering system for stress conditions (Wieczorek, 2015; Bhosale et al., 2018). In this respect, the TF E2Fe/DEL1 is an inhibitor of endoreduplication that maintains the mitotic state of proliferating cells by suppressing transcription of genes necessary to enter the endocycle (Dimova and Dyson, 2005; Inzé and De Veylder, 2006). The timing of cell cycle exit and onset of endoreduplication is determined by the levels of E2Fe/DEL1, which control anaphase-promoting activator genes such as CCCS52A2 (Lammens et al., 2008). Arabidopsis has three *DEL* genes (*DEL1*, *DEL2* and *DEL3*). Loss of DEL1 function results in increased ploidy, while ectopic expression of *DEL1* results in decreased endoreduplication levels and cells are prone to rapid expansion (Ramirez-Parra et al., 2004; Vlieghe et al., 2005; Lammens et al., 2008). The role of E2Fe/DEL in GCs formation induced by RKNs was analyzed. Ectopic expression of *DEL1* resulted in morphological changes of the GCs within the galls, as the GCs were smaller with profuse cell wall invaginations and smaller nuclei than in the wild type line at 21 dpi. Furthermore, the *DEL1* overexpressing line exhibited a significant reduction in the number of *M. incognita* egg masses due to an induction of the mitotic state, resulting in severe impairment of reproduction. Conversely, the *del1-1* loss of function line displayed small and malformed GCs with reduced mitotic activity and little cytoplasm, possibly due to a premature initiation of the endocycle (de Almeida Engler et al., 2012; Vieira et al., 2013; **Table 1**; **Figure 2**). The results indicate that multiple nuclei resulting from acytokinetic mitotic events are not sufficient to drive GC expansion. Therefore, during the plant-RKNs interaction, there is a reprogramming of the plant cell cycle machinery, inducing mitotic cycles in GCs followed by repeated endoreduplication cycles, both of which are necessary for correct GC development (de Almeida Engler et al., 2012; **Table 1**; **Figure 2**). Although only data from the variation of expression levels during RKNs infection and *in situ* localization of its transcripts are available, the orthologue *DEL1* gene from Arabidopsis in woody plants, such as *Prunus sogdiana*, *PsoDEL1*, also appears to be negatively correlated with endoreduplication and growth of GCs. *PsoDEL1* exhibited weak expression in the feeding sites during the early stages of infection (3 dpi). As the infection progressed, the hybridization signal was barely detected at the feeding site and within the GCs at 7, 14 and 21 dpi (Xiao et al., 2020; **Table 1**; **Figure 2**). Therefore, it is highly probable that the role of DEL1 is conserved in distant plant species during feeding site and GCs formation.

Interestingly, E2Fe/DEL1 also plays a role in SA biosynthesis as a transcriptional repressor of a member of the isochorismate pathway, *ENHANCED DISEASE SUSCEPTIBILITY 5 (EDS5)*, that encodes a SA transporter required for elevated SA immunity in Arabidopsis (Chandran et al., 2014). Repression of genes involved in plant defenses is a characteristic of the compatible interaction between RKNs and dicotyledonous or monocotyledonous species, such as Arabidopsis and rice, particularly in GCs (Barcala et al., 2010; Ji et al., 2013). Furthermore, the identification of *M. incognita* effectors, such as Mi-ISC-1, confirms that the nematode actively deploys a functional isochorismatase to suppress SA-mediated plant defences by altering the isochorismate synthase pathway for SA biosynthesis, favoring parasitism (Qin et al., 2022). The *del1-1* mutant showed SA accumulation in 7 dpi Arabidopsis galls, while in uninfected roots, the SA levels did not change significantly compared to the control background Col-0. Therefore, DEL1 seems to repress SA biosynthesis in RKNs-induced galls in Arabidopsis (Nakagami et al., 2020; **Table 1**; **Figure 2**). As a result, *del1-1* mutant galls at 5 dpi exhibited more intense lignin staining than wild type galls, and the expression patterns of genes encoding enzymes related to lignin biosynthesis, such as 4-coumarate: CoA ligase 1 (4CL1), 4CL2, alcohol dehydrogenase 5 (CAD5), phenylalanine ammonia-lyase 1 (PAL1), PAL2, and cinnamate 4-hydroxylase (C4H) were significantly up-regulated as compared to wild type galls (Nakagami et al., 2020). The loss of function of DEL1/E2Fe in Arabidopsis galls leads to enhanced resistance and it also causes growth inhibition, likely due to excessive lignification and/or SA accumulation in RKNs-induced galls. Therefore, DEL1 may mediate a balance between growth and defense by limiting the accumulation of SA in the infection sites (Nakagami et al., 2020), similar to what was reported during fungal infection in leaves (Chandran et al., 2014).

Plant MYB3R TFs, which are homologous to Myb oncoproteins, are also involved in controlling mitosis and cytokinesis progression by recognizing Mitotic-specific activator (MSA) elements present in genes expressed during the G2 and M-phase, such as *B-cyclins* (Ito et al., 1998, 2001; Menges et al., 2005). The MYB transcription factor family is a large family found in all eukaryotes and is involved in various processes that control plant development, metabolism, and responses to biotic and abiotic stress (Dubos et al., 2010). In Arabidopsis, five *MYB3R* genes have been identified. Among them, *MYB3R1* and *MYB3R4* function redundantly as activators, with *MYB3R4* contributing more to the activation of G2/M phase-specific genes (Haga et al., 2007; Ito et al., 2001). However, MYB3R1 has a dual function as it can act as a repressor along with MYB3R3 and MYB3R5. *MYB3R4* is only expressed during the G2/M phase, whereas the repressor-type MYB3Rs are active in post-mitotic cells and proliferative cells outside the G2/M phase (Kobayashi et al., 2015a; Kobayashi et al., 2015b). In Arabidopsis, *MYB3R4::GUS* lines showed a GUS signal in the vasculature, where a weak expression of *MYB3R5::GUS* was detected. Following *M. incognita* infection, the *MYB3R4::GUS* line exhibited GUS signal in the centre of 3, 5 and 7 dpi galls, while the *MYB3R5::GUS* line showed two strands of signal surrounding the centre of 3 and 5 dpi galls that decreased and became patchy at 7 dpi. In contrast, no signal was detected in the roots of the *MYB3R3::GUS* line, whether infected or uninfected. The loss of function lines *myb3r1*, *myb3r5* and *myb3r1/3/5* showed no effect on nematode infection. In contrast, *myb3r3*, *myb3r4*, *myb3r1/4* and *myb3r3/5* showed a significant reduction in the number of galls compared to wild type plants (Suzuki et al., 2021a; **Table 1**; **Figure 2**). It is known that the *myb3r1/4* mutant presents aberrant cytokinesis and down-regulation of cell cycle genes (Haga et al., 2007, Haga et al., 2011), and MYB3R4 is involved in endoreduplication, acting as an activator or repressor depending on its phosphorylation state (Chandran et al., 2010). In addition, MYB3Rs proteins can form complexes during cell cycle progression. For example, MYB3R4 interacts with RBR1 (Retinoblastoma-related) and E2FB, while MYB3R3 interacts with RBR1 and E2FC, which are necessary for endoreduplication (Del Pozo et al., 2002, 2006). In this respect, as mentioned before, the overexpression of a transcription factor of the DP-E2F-like family (E2Fe/DEL1) that maintains the mitotic state of proliferating cells, caused increased mitotic activity and consequent endocycle inhibition in the galls formed by RKNs. Thus, the feeding cells within the galls showed multiple nuclei and inhibited cell expansion affecting nematode development (de Almeida Engler et al., 2012, de Almeida Engler and Favery, 2011).Therefore, although a direct interaction of MYB3Rs proteins with E2F members has not yet been described in the RKN interaction, the role of MYB3Rs activators in the RKNs-interaction may be related to the regulation of key cellular processes during cell cycle progression in galls/GCs development.

One of the characteristics observed in the transcriptomes of Arabidopsis galls and micro-dissected GCs are the high number of genes included in categories such as development or hormone metabolism, both directly related (Barcala et al., 2010; Silva et al., 2022). Experimental data has confirmed that gall and GCs formation share TFs that are molecular components of transduction pathways involved in lateral root and callus formation, as well as other plant developmental processes such as tuberization, nodulation, fruit development, and flowering time (Cabrera et al., 2014b; Medina et al., 2017; Diaz-Manzano et al., 2018; Olmo et al., 2019, Olmo et al., 2020). One of the initial examples discussed is LBD16, a member of the LATERAL ORGAN BOUNDARIES-DOMAIN TF family. LBD16 is a crucial component of the auxin pathway that leads to cell divisions in the xylem pole pericycle, which are necessary for lateral root formation (Goh et al., 2019) and is also involved in pluripotency acquisition in callus cells (Liu et al., 2018). *LBD16* is activated early during nematode establishment in xylem pole pericycle cells near the nematode head and by nematode secretions in protoplast. Within the forming galls its expression was maintained till medium infection stages (11 dpi) as indicated by a promoter-GUS fusion. It also showed a crucial function during RKNs establishment, but not during the establishment of CNs as loss of function lines of LBD16 (*lbd16-*1, *35S::LBD16-SRDX*; *pLBD16::LBD16-SRDX*) showed significant less infections by *M. javanica* than the control wild type line. LBD16 is also important for the GCs and galls development, as smaller galls and less expanded GCs were observed than in Col-0 in some of those mentioned loss of function lines. Interestingly, *LBD16* is regulated by auxins in galls as also described during lateral root formation (Cabrera et al., 2014b; **Table 1**; **Figure 2**). Unexpectedly, *LBD16* was locally induced in the vascular tissue of leaves after RKNs infection, as it was proven that *M. javanica* is able to establish, induce GCs, and reproduce in *in vitro* cultured Arabidopsis leaves. LBD16 is also essential for feeding site formation in leaves, as evidenced by the inability of RKNs to establish in the *35S::LBD16-SRDX* line, which contains the *LBD16* coding sequence fused to the transcriptional repressor domain SRDX driven by the 35S promoter (Olmo et al., 2017; see **Table 1**). Thus, LBD16 appears to be a conserved molecular hub connecting developmental signals with those necessary for RKNs feeding site formation in Arabidopsis. A role for LBD16 in feeding site formation induced by CNs has not been described. However, *LBD16* is induced in a WOX11-dependent manner in the primordia of adventitious lateral roots that are promoted after *H. schchatii* infection (Willig et al., 2024). This finding connects the plant-responses to both, RKNs and CNs, to the activation of critical transducers of root developmental programs. *ABERRANT LATERAL ROOT FORMATION 4* (*ALF4*) is another gene relevant to lateral root formation and gall development. It encodes a nuclear-localized protein that is not a transcription factor. However, due to its localization and participation upstream of the auxin signaling pathways leading to lateral root formation, we are mentioning it (DiDonato et al., 2004; Bagchi et al., 2018). It is also involved in developmental processes such as vascular vessels reconnection in grafting, hormone-induced callus formation or *de novo* root organogenesis from leaf explants (Sugimoto et al., 2010; Melnyk et al., 2015). *ALF4* was induced at very early infection stages of infection by *M. javanica* (1dpi), as indicated by the activation of a *pALF4:.GUS* construct. The GUS signal increased at 4 dpi and it was maintained at medium stages of gall development (7–10 dpi), but disappeared at 14 dpi. ALF4 is necessary for the proper development of galls and GCs formed by *Meloidogyne* spp in Arabidopsis, as the mutant *alf4-1* presents aberrant galls and GCs with severe structural abnormalities that cause a dramatic reduction in the nematode’s reproduction (Olmo et al., 2019).

PUCHI, a member of the ERF Transcription factor family, is also involved in the formation of new organs such as lateral roots or floral formation (Hirota et al., 2007; Karim et al., 2009). It is activated by auxin through LBD16, controlling lateral root primordium patterning (Hirota et al., 2007; Goh et al., 2019). Interestingly, it is up-regulated in galls at early-mid stages (3, 5 and 7 dpi; Yamaguchi et al., 2017; **Table 1**; **Figure 2**) in line with its promoter activity (Suzuki et al., 2021b; **Table 1**; **Figure 2**). The expression peaked at 5 dpi, but there was no significant difference in the number of galls at 2 dpi or the number of egg masses in the mutant line *puchi-1* compared to the control wild type line. These results suggest that PUCHI does not play a significant role in nematode invasion, gall formation, or nematode reproduction (Suzuki et al., 2021b; **Table 1**; **Figure 2**). However, the function of PUCHI during nematode infection may be related to cell wall morphology. This is suggested by the observation that the *puchi-1* mutant line displayed aberrant giant cells (GCs) with dramatic protrusions and invaginations containing thick cell walls that were not present in galls from the wild type line (Suzuki et al., 2021b). Trinh et al. (2019) reported that PUCHI controls the biosynthesis pathway of very long-chain fatty acid (VLCFA) during lateral root formation. Additionally, Uemura et al. (2023) found that VLCFAs can modify the cell wall through the activation of MYB93, which regulates cell wall genes. RNAseq and promoter::GUS activation assays revealed up-regulation of genes encoding 3-KETOACYL-COA SYNTHASE 1 (KCS1) and KCS20, enzymes implicated in very-long-chain fatty acid (VLCFA) synthesis in galls. Their expression peaked at around 5 dpi, similar to that of PUCHI. Additionally, GCs from the mutants *kcs1-5* and *puchi-1* exhibited a similar phenotype with thicker walls and protuberances compared to wild-type galls. Therefore, the observed phenotype in the *puchi-1* mutant may be attributed to modifications in the VLCFA composition of the cell wall and cell membrane of the GCs (Suzuki et al., 2021b). However, PUCHI does not significantly affect nematode infectivity or reproductive parameters.

The formation of galls by RKNs is a process of post-embryonic new organogenesis as new structures specialized for nematode nourishment are induced by the nematode into the vascular cylinder of the host plant roots. The study of two TFs involved in common signaling pathways for lateral root formation, AUXIN-RESPONSIVE-FACTOR-5/ARF5, a key factor for root stem-cell niche regeneration and lateral root initiation, and GATA-TRANSCRIPTION FACTOR-23/GATA23 that specifies pluripotent founder cells during lateral root formation (De Rybel et al., 2010; De Smet et al., 2010; Efroni et al., 2016) shed light on the plant transduction pathways used or hijacked by the nematode to achieve those dramatic reprogramming events. The impact on nematode infection, galls, and GCs development was significant in the *arf5-2* mutant, as well as in inducible knockout lines for ARF5, and in a knockdown line of *GATA23* as compared to wild type lines. *pGATA23::GUS*, was induced at early infection stages, 3 dpi-7 dpi, but at 14 dpi no signal was detected, whereas *ARF5::GUS* was active in a shorter window, i.e., at 3 dpi a clear signal was detected that faded at 7 dpi, this confirmed their induction at early-mid infection according to their putative roles during galls/GCs formation (Olmo et al., 2020; **Table 1**; **Figure 2**). Therefore, the results suggest that transient pluripotency reprogramming, which leads to lateral root founder cell-like specification and root regeneration, is also necessary for gall/GCs organogenesis. In contrast, other TFs that are the main upstream transducers during lateral root development, such as ARF7 and ARF19 (Okushima et al., 2007), did not exhibit a significant role or specific expression pattern during gall/GCs formation (Olmo et al., 2020; **Table 1**; **Figure 2**). However, the regulation of another auxin TF in galls, ARF3, was also similar to that of lateral root growth (Marin et al., 2010; Cabrera et al., 2016). ARF3 is a TF that participates in a regulatory module. In this module, miR390a controls the biogenesis of TAS3-derived tasiRNAs that regulate the auxin responsive factors ARF2, ARF3 and ARF4 by degrading their transcripts and controlling lateral root growth (Marin et al., 2010). Two sensor lines (*pARF3:ARF3-GUS* and a tasiRNA-resistant ARF3 line, *pARF3:ARF3m-GUS*) indicated the binding of TAS3-derived tasiRNAs to the ARF3 sequence in galls. The results strongly suggest that the promoters of miR390 and TAS3 are active, and their products are functional in galls, repressing ARF3 (Cabrera et al., 2016; **Table 1**). Therefore, silencing of *ARF3* seems to be important during gall development and establishment of RKNs. Recently, other ARFs, such as ARF8A and ARF8B, have been studied in tomato. These were induced during the early to mid-infection stages (7-14 dpi) in galls and GCs of tomato transgenic lines *pARF8A:GUS* and *pARF8B:GUS*. The up-regulation of *ARF8A/B* transcripts in galls compared to uninfected roots in transcriptomic analysis (RNAseq) is due to the high activity of their promoter combined with reduced silencing by miR167. Furthermore, the mutant lines *slarf8a*, *slarf8ab*, and *slarf8ab* showed severely compromised infection and reproduction of *M. incognita*. In addition, expression of *ARF8A* and *ARF8B* is required for correct giant development as the former mutant lines showed giant cells significantly smaller than in the wild type line (Noureddine et al., 2023; **Table 1**; **Figure 2**). All these data, support a key role for ARF8s in feeding site formation.

Following the robust hypothesis that gall/GCs formation is a new organogenesis process, and the described similarities with callus formation, it is important to note that callus formation involves the differentiation of pericycle or pericycle-like cells in a process that resembles root tip organization. Thus, crucial root meristem (RAM) TFs marker genes, namely *SHORTROOT/SHR, SCARECROW/SCR, SCHIZORIZA/SCZ, and WUSCHELRELATED-HOMEOBOX-5/WOX5* are expressed (Sugimoto et al., 2010). It also requires the ectopic activation of a lateral root developmental program and consequently the expression of *LBD* genes (Sugimoto et al., 2010). It is noteworthy that these genes were induced very early during gall formation (2-5 dpi), but no signal was detected 7-8 dpi and most of them also played important roles in the establishment of RKNs. Thus, the activation of plant developmental programs that promote transient pluripotency/stemness leads to the generation of quiescent center and meristematic-like cell identities within the vascular cylinder of galls (Olmo et al., 2020; **Table 1**; **Figure 2**). Moreover, a process of new organogenesis also involves revascularisation, which is crucial for maintaining GCs growth as they are symplastically isolated specialized transfer cells (Hoth et al., 2008; Rodiuc et al., 2014). Phloem formation is induced during gall development (Bartlem et al., 2014). APL (Altered phloem development), a MYB coiled-coil-type TF involved in phloem identity acquisition, is expressed in protophloem, metaphloem and companion cells (Bonke et al., 2003). It is induced early after infection with *M. incognita* and *M. javanica* in Arabidopsis, as shown by an A*PL::GUS* line with a strand signal in 3 dpi galls that increases at 5 dpi (Suzuki et al., 2021a; **Table 1**; **Figure 2**). However, functional tests are still needed to confirm its role during gall formation.

It is known that the balance between cell proliferation and cell differentiation in the procambium is regulated downstream of the receptor and kinase cascade by the WOX4 TF. Cyst nematodes have been shown to modulate the procambial cell proliferation of feeding cell formation probably by mimicking the plant B-type CLE TDIF (tracheary element differentiation inhibitory factor) peptide that is encoded by two genes *CLE41* and *CLE44* in Arabidopsis, and by taking control of the (TDIF RECEPTOR/PHLOEM INTERCALATED WITH XYLEM (TDR/PXY)-WOX4 signaling pathway (Guo et al., 2017). In this regard, *WOX4* and *ATHB8*, typical procambium marker genes, were induced in *M. incognita* galls at 3, 5 and 7 dpi. However, the analysis of *athb8-11* and *wox4-1* loss-of-function mutants did not cause any visible effect on the infection parameters or gall diameter. These genes usually function redundantly; therefore, single mutations were probably not sufficient to prevent procambial cell formation (Yamaguchi et al., 2017; **Table 1**; **Figure 2**). Nevertheless, the connection between gall formation and different developmental pathways is evident. The riboregulator miRNA172 post-transcriptionally targets a small group of regulatory repressor genes, including *APETALA2* (*AP2*) and AP2-like genes, such as *TARGET OF EARLY ACTIVATION TAGGED 1* (*TOE1*). These miRNA172-targeted AP2-like TFs are involved in controlling several developmental processes, such as plant aging, flowering time, tuber formation, fruit growth, and nodulation (Martin et al., 2009; Zhu and Helliwell, 2010; Yan et al., 2013; Wang et al., 2014; Ripoll et al., 2015). Functional analysis of RKNs infective and reproductive parameters was conducted on Arabidopsis lines with altered activities based on *35S::MIMICRY172 (MIM172)*, *35S::TARGET OF EARLY ACTIVATION TAGGED 1* (*TOE1*)-*miR172-resistant* (*35S::TOE1R*) and mutant (flowering locus T-10 (*ft-10*)) during gall and GCs development. The results indicated that the regulatory module miRNA172/TOE1/FT plays a crucial role during GCs and gall development (Diaz-Manzano et al., 2018; **Table 1**; **Figure 2**). Therefore, the repression of *TOE1* by miRNA172 is relevant for the normal establishment of RKNs and the formation of galls/GCs.

Interestingly, the SPL7/MIR408-UCC2/MIR398-CSD1 copper module (Griffiths-Jones et al., 2007) is also functional and active within galls (Noureddine et al., 2022; **Table 1**; **Figure 2**). Loss of function lines of miR398b/c and miR408 in Arabidopsis, resulted in fewer galls and smaller infection sites as compared with the control lines. These findings together with the expression data of two microRNA families, *miR398* and *miR408*, upregulated in galls, similarly to that of the TF SLP7, strongly suggest that the expression of *MIR408* and *MIR398B* and *-C* is activated by SPL7 in response to a decrease in copper concentration in galls (Noureddine et al., 2022). The role of this module might be related to its involvement in lignin metabolism (Reyt et al., 2020) as the cell wall suffer dramatic changes during gall and GCs development and numerous cell wall modifying enzymes are activated (Wieczorek, 2015). However, further research will be needed to elucidate it.

## Conclusion

Despite the abundance of DEGs encoding plant TFs during the RKNs interaction in several plant species, that for example in the Arabidopsis transcriptomes cover most of the TFs families identified within the genome (**Figure 1**), their biological function during RKNs infection is still poorly understood. The functional role of only around 40 TFs have been assessed (**Table 1**). Most of the data was obtained from plant lines with altered expression for each TF, mainly loss of function lines, that were infected, and significant differences either in the infection rate, gall formation, gall or GCs development compared to their control wild type lines were encountered. As a result, a clear phenotype during RKNs infection was identified. Two main groups of functionalities can be identified: TFs related to plant defenses, whose downstream targets are defense-related genes (see **Table 1**; **Figure 2**), and TFs involved in plant developmental programs, such as lateral root and/or callus formation, root apical meristem, or root regeneration. These are presumably hijacked by nematodes for their own benefit, including some TFs with a role in basic cellular functions, such as cell cycle control (**Table 1**; **Figure 2**). Interestingly, there is increasing evidence of the involvement of TFs with a dual role in plant development and defense and/or as integrator hubs between stress signals and developmental signals, such as DEL1, ERF109, ERF115, ERF114 and ERF6 (**Table 1**; **Figure 2**). However, the signal transduction pathways regulated by those TFs during RKNs infection are mostly unknown or only partially understood. Nevertheless, few regulatory modules involving TFs have been fully or partially proven in the interaction between RKNs and plants. These include the miRNA172/TOE1/FT module (Diaz-Manzano et al., 2018) and the SPL7/MIR408-UCC2/MIR398-CSD1 copper module (Noureddine et al., 2022). Clearly, further studies are needed to increase our knowledge in the regulatory networks driven by plant TFs modified by the nematode and as a plant response during the RKN interactions.

## Author contributions

JD-F: Conceptualization, Data curation, Validation, Visualization, Writing – original draft, Writing – review & editing, Formal analysis, Investigation, Methodology, Software. AG-R: Investigation, Methodology, Writing – original draft. CE: Conceptualization, Writing – original draft, Data curation, Funding acquisition, Project administration, Resources, Supervision, Validation, Visualization, Writing – review & editing.

## References

[B1] Absmanner B. Stadler R. Hammes U. Z. (2013). Phloem development in nematode-induced feeding sites: The implications of auxin and cytokinin. Front. Plant Sci. 4, 241. doi: 10.3389/fpls.2013.00241, PMID: 23847644 PMC3703529

[B2] Ali M. A. Wieczorek K. Kreil D. P. Bohlmann H. (2014). The beet cyst nematode Heterodera schachtii modulates the expression of WRKY transcription factors in syncytia to favour its development in Arabidopsis roots. PloS One 9, e102360. doi: 10.1371/journal.pone.0102360, PMID: 25033038 PMC4102525

[B3] Ambawat S. Sharma P. Yadav N. R. Yadav R. C. (2013). MYB transcription factor genes as regulators for plant responses: an overview. Physiol. Mol. Biol. Plants 19, 307–321. doi: 10.1007/s12298-013-0179-1, PMID: 24431500 PMC3715649

[B4] Atamian H. S. Eulgem T. Kaloshian I. (2012). SlWRKY70 is required for Mi-1-mediated resistance to aphids and nematodes in tomato. Planta 235, 299–309. doi: 10.1007/s00425-011-1509-6, PMID: 21898085

[B5] Bagchi R. Melnyk C. W. Christ G. Winkler M. Kirchsteiner K. Salehin M.. (2018). The Arabidopsis ALF4 protein is a regulator of SCF E3 ligases. EMBO J. 37, 255–268. doi: 10.15252/embj.201797159, PMID: 29233834 PMC5770881

[B6] Barcala M. García A. Cabrera J. Casson S. Lindsey K. Favery B.. (2010). Early transcriptomic events in microdissected Arabidopsis nematode-induced giant cells. Plant J. 61, 698–712. doi: 10.1111/tpj.2010.61.issue-4, PMID: 20003167

[B7] Bartlem D. G. Jones M. G. K. Hammes U. Z. (2014). Vascularization and nutrient delivery at root-knot nematode feeding sites in host roots. In J. Exp. Bot. 65, 1789–1798. doi: 10.1093/jxb/ert415, PMID: 24336493

[B8] Bhattarai K. K. Atamian H. S. Kaloshian I. Eulgem T. (2010). WRKY72-type transcription factors contribute to basal immunity in tomato and Arabidopsis as well as gene-for-gene resistance mediated by the tomato R gene Mi-1. Plant J. 63, 229–240. doi: 10.1111/j.1365-313X.2010.04232.x, PMID: 20409007

[B9] Bhosale R. Boudolf V. Cuevas F. Lu R. Eekhout T. Hu Z.. (2018). A spatiotemporal DNA endoploidy map of the arabidopsis root reveals roles for the endocycle in root development and stress adaptation. Plant Cell 30, 2330–2351. doi: 10.1105/tpc.17.00983, PMID: 30115738 PMC6241279

[B10] Birkenbihl R. P. Liu S. Somssich I. E. (2017). Transcriptional events defining plant immune responses. Curr. Opin. Plant Biol. 38, 1–9. doi: 10.1016/j.pbi.2017.04.004, PMID: 28458046

[B11] Bonke M. Thitamadee S. Mähönen A. P. Hauser M.-T. Helariutta Y. (2003). APL regulates vascular tissue identity in Arabidopsis. Nature 426, 181–186. doi: 10.1038/nature02100, PMID: 14614507

[B12] Cabrera J. Barcala M. García A. Rio-Machín A. Medina C. Jaubert-Possamai S.. (2016). Differentially expressed small RNAs in Arabidopsis galls formed by Meloidogyne javanica: a functional role for miR390 and its TAS3-derived tasiRNAs. New Phytol. 209, 1625–1640. doi: 10.1111/nph.13735, PMID: 26542733

[B13] Cabrera J. Bustos R. Favery B. Fenoll C. Escobar C. (2014a). NEMATIC: A simple and versatile tool for the in silico analysis of plant–nematode interactions. Mol. Plant Pathol. 15, 627–636. doi: 10.1111/mpp.12114, PMID: 24330140 PMC6638708

[B14] Cabrera J. Diaz-Manzano F. E. Saínchez M. Rosso M. N. Melillo T. Goh T.. (2014b). A role for LATERAL ORGAN BOUNDARIES-DOMAIN 16 during the interaction Arabidopsis-Meloidogyne spp. provides a molecular link between lateral root and root-knot nematode feeding site development. New Phytol. 203, 632–645. doi: 10.1111/nph.12826, PMID: 24803293

[B15] Chandran D. Inada N. Hather G. Kleindt C. K. Wildermuth M. C. (2010). Laser microdissection of Arabidopsis cells at the powdery mildew infection site reveals site-specific processes and regulators. Proc. Natl. Acad. Sci. United States America 107, 460–465. doi: 10.1073/pnas.0912492107, PMID: 20018666 PMC2806765

[B16] Chandran D. Rickert J. Huang Y. Steinwand M. A. Marr S. K. Wildermuth M. C. (2014). Atypical E2F transcriptional repressor DEL1 acts at the intersection of plant growth and immunity by controlling the hormone salicylic acid. Cell Host Microbe 9;15, 506–513. doi: 10.1016/j.chom.2014.03.007, PMID: 24721578

[B17] Chinnapandi B. Bucki P. Braun-Miyara S. (2017). SlWRKY45, nematode-responsive tomato WRKY gene, enhances susceptibility to the root knot nematode; M. javanica infection. Plant Signaling Behav. 2, 12(12). doi: 10.1080/15592324.2017.1356530, PMID: 29271721 PMC5792125

[B18] Chinnapandi B. Bucki P. Fitoussi N. Kolomiets M. Borrego E. Braun-Miyara S. (2019). Tomato SlWRKY3 acts as a positive regulator for resistance against the root-knot nematode Meloidogyne javanica by activating lipids and hormone-mediated defense-signaling pathways. Plant Signaling Behav. 14, 1601951. doi: 10.1080/15592324.2019.1601951, PMID: 31010365 PMC6546140

[B19] de Almeida Engler J. Favery B. (2011). “The plant cytoskeleton remodelling in nematode induced feeding sites,” in Genomics and molecular genetics of plant-nematode interactions (London, UK: Springer Science & Business Media, B.V.), 369–393. doi: 10.1007/978-94-007-0434-3_18

[B20] de Almeida Engler J. De Vleesschauwer V. Burssens S. Celenza J. L. Jr. Inzé D. Van Montagu M.. (1999). Molecular markers and cell cycle inhibitors show the importance of cell cycle progression in nematode-induced galls and syncytia. Plant Cell 11, 793–808. doi: 10.1105/tpc.11.5.793, PMID: 10330466 PMC144216

[B21] de Almeida Engler J. Kyndt T. Vieira P. Van Cappelle E. Boudolf V. Sanchez V.. (2012). CCS52 and DEL1 genes are key components of the endocycle in nematode-induced feeding sites. Plant J. 72, 185–198. doi: 10.1111/j.1365-313X.2012.05054.x, PMID: 22640471

[B22] Del Pozo J. C. Boniotti M. B. Gutierrez C. (2002). Arabidopsis E2Fc functions in cell division and is degraded by the ubiquitin-SCFAtSKP2 pathway in response to light. Plant Cell 14, 3057–3071. doi: 10.1105/tpc.006791, PMID: 12468727 PMC151202

[B23] Del Pozo J. C. Diaz-Trivino S. Cisneros N. Gutierrez C. (2006). The balance between cell division and endoreplication depends on E2FC-DPB, transcription factors regulated by the ubiquitin-SCFSKP2A pathway in Arabidopsis. Plant Cell 18, 2224–2235. doi: 10.1105/tpc.105.039651, PMID: 16920782 PMC1560920

[B24] De Meutter J. Tytgat T. Witters E. Gheysen G. Van Onckelen H. Gheysen G. (2003). Identification of cytokinins produced by the plant parasitic nematodes Heterodera schachtii and Meloidogyne incognita. Mol. Plant Pathol. 4, 271–277. doi: 10.1046/j.1364-3703.2003.00176.x, PMID: 20569387

[B25] De Rybel B. Vassileva V. Parizot B. Demeulenaere M. Grunewald W. Audenaert D.. (2010). A novel aux/IAA28 signaling cascade activates GATA23-dependent specification of lateral root founder cell identity. Curr. Biol. 12;20, 1697–1706. doi: 10.1016/j.cub.2010.09.007, PMID: 20888232

[B26] De Smet I. Lau S. Voss U. Vanneste S. Benjamins R. Rademacher E. H.. (2010). Bimodular auxin response controls organogenesis in Arabidopsis. Proc. Natl. Acad. Sci. U.S.A. 9;107, 2705–2710. doi: 10.1073/pnas.0915001107, PMID: 20133796 PMC2823897

[B27] Diaz-Manzano F. E. Cabrera J. Ripoll J. J. Del Olmo I. Andrés M. F. Silva A. C.. (2018). A role for the gene regulatory module microrna172/TARGET OF EARLY ACTIVATION TAGGED 1/FLOWERING LOCUS T (Mirna172/TOE1/FT) in the feeding sites induced by meloidogyne javanica in arabidopsis thaliana. New Phytol. 217, 813–827. doi: 10.1111/nph.14839, PMID: 29105090 PMC5922426

[B28] DiDonato R. J. Arbuckle E. Buker S. Sheets J. Tobar J. Totong R.. (2004). Arabidopsis ALF4 encodes a nuclear-localized protein required for lateral root formation. Plant J. 37, 340–353. doi: 10.1046/j.1365-313X.2003.01964.x, PMID: 14731255

[B29] Dimova D. K. Dyson N. J. (2005). The E2F transcriptional network: Old acquaintances with new faces. Oncogene 24, 2810–2826. doi: 10.1038/sj.onc.1208612, PMID: 15838517

[B30] Dubos C. Stracke R. Grotewold E. Weisshaar B. Martin C. Lepiniec L. (2010). MYB transcription factors in Arabidopsis. Trends Plant Sci. 15, 573–581. doi: 10.1016/j.tplants.2010.06.005, PMID: 20674465

[B31] Efroni I. Mello A. Nawy T. Ip P. L. Rahni R. DelRose N.. (2016). Root regeneration triggers an embryo-like sequence guided by hormonal interactions. Cell 16;165, 1721–1733. doi: 10.1016/j.cell.2016.04.046, PMID: 27212234 PMC4912400

[B32] Elling A. A. (2013). Major emerging problems with minor Meloidogyne species. Phytopathology 103, 1092–1102. doi: 10.1094/PHYTO-01-13-0019-RVW, PMID: 23777404

[B33] Escobar C. Barcala M. Cabrera J. Fenoll C. (2015). Overview of root-knot nematodes and giant cells. Adv. Botanical Res. 73, 1–32. doi: 10.1016/bs.abr.2015.01.001

[B34] Frerigmann H. Gigolashvili T. (2014). MYB34, MYB51, and MYB122 distinctly regulate indolic glucosinolate biosynthesis in Arabidopsis thaliana. Mol. Plant 7, 814–828. doi: 10.1093/mp/ssu004, PMID: 24431192

[B35] Fuller V. L. Lilley C. J. Atkinson H. J. Urwin P. E. (2007). Differential gene expression in Arabidopsis following infection by plant-parasitic nematodes Meloidogyne incognita and Heterodera schachtii. Mol. Plant Pathol. 8, 595–609. doi: 10.1111/j.1364-3703.2007.00416.x, PMID: 20507524

[B36] Gheysen G. Fenoll C. (2011). “Arabidopsis as a tool for the study of plant–nematode interactions,” in Genomics and molecular genetics of plant–nematode interactions. Eds. Jones J. Gheysen G. Fenoll C. (Springer, Dordrecht, the Netherlands), 139–156.

[B37] Goh T. Toyokura K. Yamaguchi N. Okamoto Y. Uehara T. Kaneko S.. (2019). Lateral root initiation requires the sequential induction of transcription factors LBD16 and PUCHI in Arabidopsis thaliana. New Phytol. 224, 749–760. doi: 10.1111/nph.16065, PMID: 31310684

[B38] Griffiths-Jones S. Saini H. K. Van Dongen S. Enright A. J. (2007). miRBase: tools for microRNA genomics. Nucleic Acids Res. 36, D154–D158. doi: 10.1093/nar/gkm952, PMID: 17991681 PMC2238936

[B39] Guarneri N. Willig J. J. Sterken M. G. Zhou W. Hasan M. S. Sharon L.. (2022). Root architecture plasticity in response to endoparasitic cyst nematodes is mediated by damage signaling. New Phytol. 237, 807–822. doi: 10.1111/nph.18570, PMID: 36285401 PMC10108316

[B40] Guo X. Wang J. Gardner M. Fukuda H. Kondo Y. Etchells J. P.. (2017). Identification of cyst nematode B-type CLE peptides and modulation of the vascular stem cell pathway for feeding cell formation. PloS Pathog. 13, e1006142. doi: 10.1371/journal.ppat.1006142, PMID: 28158306 PMC5319780

[B41] Haga N. Kato K. Murase M. Araki S. Kubo M. Demura T.. (2007). R1R2R3-Myb proteins positively regulate cytokinesis through activation of KNOLLE transcription in Arabidopsis thaliana. Development 134, 1101–1110. doi: 10.1242/dev.02801, PMID: 17287251

[B42] Haga N. Kobayashi K. Suzuki T. Maeo K. Kubo M. Ohtani M.. (2011). Mutations in MYB3R1 and MYB3R4 cause pleiotropic developmental defects and preferential down-regulation of multiple G2/M-specific genes in Arabidopsis. Plant Physiol. 157, 706–717. doi: 10.1104/pp.111.180836, PMID: 21862669 PMC3192584

[B43] Hirota A. Kato T. Fukaki H. Aida M. Tasaka M. (2007). The auxin-regulated AP2/EREBP gene PUCHI is required for morphogenesis in the early lateral root primordium of arabidopsis. Plant Cell 19, 2156–2168. doi: 10.1105/tpc.107.050674, PMID: 17630277 PMC1955702

[B44] Hoth S. Stadler R. Sauer N. Hammes U. Z. Beachy R. N. Danforth D. (2008). Differential vascularization of nematode-induced feeding sites. Proc. Natl. Acad. Sci. 105, 12617–12622. doi: 10.1073/pnas.0803835105, PMID: 18711135 PMC2527960

[B45] Huang H. Zhao W. Qiao H. Li C. Sun L. Yang R.. (2022). SlWRKY45 interacts with jasmonate-ZIM domain proteins to negatively regulate defense against the root-knot nematode Meloidogyne incognita in tomato. Hortic. Res. 5;9, uhac197. doi: 10.22541/au.164151237.72268573/v1, PMID: 36338841 PMC9630973

[B46] Ikeuchi M. Favero D. S. Sakamoto Y. Iwase A. Coleman D. Rymen B.. (2019). Molecular mechanisms of plant regeneration. Annu. Rev. Plant Biol. 70, 377–406. doi: 10.1146/annurev-arplant-050718-100434, PMID: 30786238

[B47] Inzé D. De Veylder L. (2006). Cell cycle regulation in plant development. Annu. Rev. Genet. 40, 77–105. doi: 10.1146/annurev.genet.40.110405.090431, PMID: 17094738

[B48] Isah T. (2019). Stress and defense responses in plant secondary metabolites production. Biol. Res. 52, 39. doi: 10.1186/s40659-019-0246-3, PMID: 31358053 PMC6661828

[B49] Ito M. Araki S. Matsunaga S. Itoh T. Nishihama R. Machida Y.. (2001). G2/M-Phase-Specific Transcription during the Plant Cell Cycle Is Mediated by c-Myb-Like Transcription Factors. Plant Cell 13, 1891–1905. doi: 10.1105/tpc.010102, PMID: 11487700 PMC139135

[B50] Ito M. Iwase M. Kodama H. Lavisse P. Komamine A. Nishihama R.. (1998). A novel cis-acting element in promoters of plant B-type cyclin genes activates M phase-specific transcription. Plant Cell 10, 331–341. doi: 10.1105/tpc.10.3.331, PMID: 9501108 PMC144003

[B51] Jammes F. Lecomte P. de Almeida-Engler J. Bitton F. Martin-Magniette M. L. Renou J. P.. (2005). Genome-wide expression profiling of the host response to root-knot nematode infection in Arabidopsis. Plant J. 44, 447–458. doi: 10.1111/j.1365-313X.2005.02532.x, PMID: 16236154

[B52] Ji H. Gheysen G. Denil S. Lindsey K. Topping J. F. Nahar K.. (2013). Transcriptional analysis through RNA sequencing of giant cells induced by Meloidogyne graminicola in rice roots. J. Exp. Bot. 64, 3885–3898. doi: 10.1093/jxb/ert219, PMID: 23881398 PMC3745741

[B53] Jiang J. Ma S. Ye N. Jiang M. Cao J. Zhang J. (2017). WRKY transcription factors in plant responses to stresses. J. Integr. Plant Biol. 59, 86–101. doi: 10.1111/jipb.12513, PMID: 27995748

[B54] Jin J. P. Zhang H. Kong L. Gao G. Luo J. C. (2014). PlantTFDB 3.0: a portal for the functional and evolutionary study of plant transcription factors. Nucleic Acids Res. 42, D1182–D1187. doi: 10.1093/nar/gkt1016, PMID: 24174544 PMC3965000

[B55] Karim M. R. Hirota A. Kwiatkowska D. Tasaka M. Aida M. (2009). A role for arabidopsis PUCHI in floral meristem identity and bract suppression. Plant Cell 21, 1360–1372. doi: 10.1105/tpc.109.067025, PMID: 19482972 PMC2700531

[B56] Kikuchi T. Eves-van den Akker S. Jones J. T. (2017). Genome evolution of plant-parasitic nematodes. Annu. Rev. Phytopathol. 55, 333–354. doi: 10.1146/annurev-phyto-080516-035434, PMID: 28590877

[B57] Knoth C. Ringler J. Dangl J. L. Eulgem T. (2007). Arabidopsis WRKY70 is required for full RPP4-mediated disease resistance and basal defense against Hyaloperonospora parasitica. Mol. Plant Microbe Interact. 20, 120–128. doi: 10.1094/MPMI-20-2-0120, PMID: 17313163

[B58] Kobayashi K. Suzuki T. Iwata E. Magyar Z. Bögre L. Ito M. (2015a). MYB3Rs, plant homologs of Myb oncoproteins, control cell cycle-regulated transcription and form DREAM-like complexes. Transcription 6, 106–111. doi: 10.1080/21541264.2015.1109746, PMID: 26556011 PMC4802795

[B59] Kobayashi K. Suzuki T. Iwata E. Nakamichi N. Suzuki T. Chen P.. (2015b). Transcriptional repression by MYB3R proteins regulates plant organ growth. EMBO J. 34, 1992–2007. doi: 10.15252/embj.201490899, PMID: 26069325 PMC4551348

[B60] Kumar A. Sichov N. Bucki P. Miyara S. B. (2023). SlWRKY16 and SlWRKY31 of tomato, negative regulators of plant defense, involved in susceptibility activation following root-knot nematode Meloidogyne javanica infection. Sci. Rep. 5;13, 14592. doi: 10.1038/s41598-023-40557-z, PMID: 37669955 PMC10480479

[B61] Kyndt T. Denil S. Haegeman A. Trooskens G. Bauters L. Van Criekinge W.. (2012). Transcriptional reprogramming by root knot and migratory nematode infection in rice. New Phytol. 196, 887–900. doi: 10.1111/j.1469-8137.2012.04311.x, PMID: 22985291

[B62] Lammens T. Boudolf V. Kheibarshekan L. Zalmas L. P. Gaamouche T. Maes S.. (2008). Atypical E2F activity restrains APC/CCCS52A2 function obligatory for endocycle onset. Proc. Natl. Acad. Sci. U.S.A. 23;105, 14721–14726. doi: 10.1073/pnas.0806510105, PMID: 18787127 PMC2567204

[B63] Li J. Brader G. Kariola T. Tapio Palva E. (2006). WRKY70 modulates the selection of signaling pathways in plant defense. Plant J. 46, 477–491. doi: 10.1111/j.1365-313X.2006.02712.x, PMID: 16623907

[B64] Liu J. Hu X. Qin P. Prasad K. Hu Y. Xu L. (2018). The WOX11-LBD16 pathway promotes pluripotency acquisition in callus cells during *de novo* shoot regeneration in tissue culture. Plant Cell Physiol. 59, 734–743. doi: 10.1093/pcp/pcy010, PMID: 29361138

[B65] Marin E. Jouannet V. Herz A. Lokerse A. S. Weijers D. Vaucheret H.. (2010). miR390, Arabidopsis TAS3 tasiRNAs, and their AUXIN RESPONSE FACTOR targets define an autoregulatory network quantitatively regulating lateral root growth. Plant Cell 22, 1104–1117. doi: 10.1105/tpc.109.072553, PMID: 20363771 PMC2879756

[B66] Martin A. Adam H. Diaz-Mendoza M. Zurczak M. Gonzalez-Schain N. D. Suarez-Lopez P. (2009). Graft-transmissible induction of potato tuberization by the microRNA miR172. Development 136, 2873–2881. doi: 10.1242/dev.031658, PMID: 19666819

[B67] Medina C. da Rocha M. Magliano M. Ratpopoulo A. Revel B. Marteu N.. (2017). Characterization of microRNAs from Arabidopsis galls highlights a role for miR159 in the plant response to the root-knot nematode Meloidogyne incognita. New Phytol. 216, 882–896. doi: 10.1111/nph.14717, PMID: 28906559

[B68] Melnyk C. W. Schuster C. Leyser O. Meyerowitz E. M. (2015). ). A developmental framework for graft formation and vascular reconnection in Arabidopsis thaliana. Curr. Biol. 25, 1306–1318. doi: 10.1016/j.cub.2015.03.032, PMID: 25891401 PMC4798781

[B69] Meng X. Xu J. He Y. Yang K. Y. Mordorski B. Liu Y.. (2013). Phosphorylation of an ERF transcription factor by Arabidopsis MPK3/MPK6 regulates plant defense gene induction and fungal resistance. Plant Cell 25, 1126–1142. doi: 10.1105/tpc.112.109074, PMID: 23524660 PMC3634681

[B70] Menges M. De Jager S. M. Gruissem W. Murray J. A. H. (2005). Global analysis of the core cell cycle regulators of Arabidopsis identifies novel genes, reveals multiple and highly specific profiles of expression and provides a coherent model for plant cell cycle control. Plant J. 41, 546–566. doi: 10.1111/j.1365-313X.2004.02319.x, PMID: 15686519

[B71] Nakagami S. Saeki K. Toda K. Ishida T. Sawa S. (2020). The atypical E2F transcription factor DEL1 modulates growth–defense tradeoffs of host plants during root-knot nematode infection. Sci. Rep. 1;10, 8836. doi: 10.1038/s41598-020-65733-3, PMID: 32483126 PMC7264364

[B72] Nakano T. Suzuki K. Fujimura T. Shinshi H. (2006). Genome-wide analysis of the ERF gene family in arabidopsis and rice. Plant Physiol. 140, 411–432. doi: 10.1104/pp.105.073783, PMID: 16407444 PMC1361313

[B73] Ngou B. P. M. Ahn H. K. Ding P. Jones J. D. G. (2020). Mutual potentiation of plant immunity by cell-surface and intracellular receptors. Nature 592, 110–115. doi: 10.1038/s41586-021-03315-7, PMID: 33692545

[B74] Nie W. Liu L. Chen Y. Luo M. Feng C. Wang C.. (2023). Identification of the Regulatory Role of SlWRKYs in Tomato Defense against Meloidogyne incognita. Plants (Basel) 22;12, 2416. doi: 10.3390/plants12132416, PMID: 37446977 PMC10346644

[B75] Noureddine Y. da Rocha M. An J. Médina C. Mejias J. Mulet K.. (2023). AUXIN RESPONSIVE FACTOR8 regulates development of the feeding site induced by root-knot nematodes in tomato. J. Exp. Bot. 29;74, 5752–5766. doi: 10.1093/jxb/erad208283-295, PMID: 37310189

[B76] Noureddine Y. Mejias J. da Rocha M. Thomine S. Quentin M. Abad P.. (2022). Copper microRNAs modulate the formation of giant feeding cells induced by the root knot nematode Meloidogyne incognita in Arabidopsis thaliana. New Phytol. 236, 283–297. doi: 10.1111/nph.18362, PMID: 35801827

[B77] Okushima Y. Fukaki H. Onoda M. Theologis A. Tasaka M. (2007). ARF7 andARF19 regulate lateral root formation via direct activation of LBD/ASL genes in Arabidopsis. Plant Cell 19, 118–130. doi: 10.1105/tpc.106.047761, PMID: 17259263 PMC1820965

[B78] Olmo R. Cabrera J. Diaz-Manzano F. E. Ruiz-Ferrer V. Barcala M. Ishida T.. (2020). Root-knot nematodes induce gall formation by recruiting developmental pathways of post-embryonic organogenesis and regeneration to promote transient pluripotency. New Phytol. 227, 200–215. doi: 10.1111/nph.16521, PMID: 32129890

[B79] Olmo R. Cabrera J. Fenoll C. Escobar C. (2019). A role for ALF4 during gall and giant cell development in the biotic interaction between Arabidopsis and Meloidogyne spp. Physiol. Plant 165, 17–28. doi: 10.1111/ppl.12734, PMID: 29573275

[B80] Olmo R. Cabrera J. Moreno-Risueno M. A. Fukaki H. Fenoll C. Escobar C. (2017). Molecular transducers from roots are triggered in arabidopsis leaves by root-knot nematodes for successful feeding site formation: A conserved post-embryogenic *de novo* organogenesis program? Front. Plant Sci. 26:8. doi: 10.3389/fpls.2017.00875, PMID: 28603536 PMC5445185

[B81] Peng Y. van Wersch R. Zhang Y. (2018). Convergent and divergent signaling in PAMP-triggered immunity and effector-triggered immunity. Mol. Plant-Microbe Interact. 31, 403–409. doi: 10.1094/MPMI-06-17-0145-CR, PMID: 29135338

[B82] Portillo M. Cabrera J. Lindsey K. Topping J. Andrés M. F. Emiliozzi M.. (2013). Distinct and conserved transcriptomic changes during nematode-induced giant cell development in tomato compared with Arabidopsis: a functional role for gene repression. New Phytol. 197, 1276–1290. doi: 10.1111/nph.12121, PMID: 23373862

[B83] Qin X. Xue B. Tian H. Fang C. Yu J. Chen C.. (2022). An unconventionally secreted effector from the root knot nematode Meloidogyne incognita, Mi-ISC-1, promotes parasitism by disrupting salicylic acid biosynthesis in host plants. Mol. Plant Pathol. 23, 516–529. doi: 10.1111/mpp.13175, PMID: 34923729 PMC8916211

[B84] Ramirez-Parra E. López-Matas M. A. Fründt C. Gutierrez C. (2004). Role of an atypical E2Ftranscription factor in the control of Arabidopsis cell growth and differentiation. Plant Cell 16, 2350–2363. doi: 10.1105/tpc.104.023978, PMID: 15308755 PMC520938

[B85] Reyt G. Chao Z. Flis P. Salas-Gonzalez I. Castrillo G. Chao D. Y.. (2020). Uclacyanin proteins are required for lignified nanodomain formation within casparian strips. Curr. Biol. 19;30, 4103–4111.e6. doi: 10.1016/j.cub.2020.07.095, PMID: 32857976 PMC7575197

[B86] Ribeiro D. G. Mota A. P. Z. Santos I. R. Arraes F. B. M. Grynberg P. Fontes W.. (2022). NBS-LRR-WRKY genes and protease inhibitors (PIs) seem essential for cowpea resistance to root-knot nematode. J. Proteomics 15;261, 104575. doi: 10.1016/j.jprot.2022.104575, PMID: 35351660

[B87] Ribeiro C. de Melo B. P. Lourenço-Tessutti I. T Ballesteros H. F. Ribeiro K. V. G. Menuet K.. (2024). MicroRNA regulation of fruit growthThe regeneration conferring transcription factor complex ERF115-PAT1 coordinates a wound-induced response in root-knot nematode induced galls. New Phytol. 241, 878–895. doi: 10.1111/nph.19399, PMID: 38044565

[B88] Ripoll J. J. Bailey L. J. Mai Q. A. Wu S. L. Hon C. T. Chapman E. J.. (2015). MicroRNA regulation of fruit growth. Nat. Plants 30;1, 15036. doi: 10.1038/nplants.2015.36, PMID: 27247036

[B89] Rodiuc N. Vieira P. Banora M. Y. de Almeida Engler J. (2014). On the track of transfer cell formation by specialized plant-parasitic nematodes. Front. Plant Sci. 5;5. doi: 10.3389/fpls.2014.00160, PMID: 24847336 PMC4017147

[B90] Rutter W. B. Franco J. Gleason C. (2022). Rooting out the mechanisms of root-knot nematode-plant interactions. Annu. Rev. Phytopathol. 60, 43-76. doi: 10.1146/annurev-phyto-021621-120943, PMID: 35316614

[B91] Sabelli P. A. Nguyen H. Larkins B. A. (2007). “Cell cycle and endosperm development,” in Annual plant reviews, volume 32: cell cycle control and plant development. Ed. Inzé E. ,. D. (Blackwell Publishing Ltd, Oxford), 294–310.

[B92] Sakuma Y. Liu Q. Dubouzet J. G. Abe H. Shinozaki K. Yamaguchi-Shinozaki K. (2002). DNA-binding specificity of the ERF/AP2 domain of Arabidopsis DREBs, transcription factors involved in dehydration- and cold-inducible gene expression. Biochem. Biophys. Res. Commun. 290, 998–1009. doi: 10.1006/bbrc.2001.6299, PMID: 11798174

[B93] Siddique S. Radakovic Z. S. de la Torre C. M. Chronis D. Novák O. Ramireddy E.. (2015). A parasitic nematode releases cytokinin that controls cell division and orchestrates feeding site formation in host plants. PNAS,112 41), 12669–12674. doi: 10.1073/pnas.1503657112, PMID: 26417108 PMC4611629

[B94] Silva A. C. Ruiz-Ferrer V. Müller S. Y. Pellegrin C. Abril-Urías P. Martínez-Gómez Á.. (2022). The DNA methylation landscape of the root-knot nematode-induced pseudo-organ, the gall, in Arabidopsis, is dynamic, contrasting over time, and critically important for successful parasitism. New Phytol. 236, 1888–1907. doi: 10.1111/nph.18395, PMID: 35872574 PMC9825882

[B95] Singh S. Singh B. Singh A. P. (2015). Nematodes: A threat to sustainability of agriculture. Proc. Environ. Sci. 29, 215–216. doi: 10.1016/j.proenv.2015.07.270

[B96] Sugimoto K. Jiao Y. Meyerowitz E. M. (2010). Arabidopsis regeneration from multiple tissues occurs via a root development pathway. Dev. Cell 16;18, 463–471. doi: 10.1016/j.devcel.2010.02.004, PMID: 20230752

[B97] Suzuki R. Ueda T. Wada T. Ito M. Ishida T. Sawa S. (2021a). Identification of genes involved in meloidogyne incognita-induced gall formation processes in arabidopsis thaliana. Plant Biotechnol. 38, 1–8. doi: 10.5511/plantbiotechnology.20.0716a, PMID: 34177318 PMC8215457

[B98] Suzuki R. Yamada M. Higaki T. Aida M. Kubo M. Tsai A. Y. L.. (2021b). PUCHI regulates giant cell morphology during root-knot nematode infection in arabidopsis thaliana. Front. Plant Sci. 12. doi: 10.3389/fpls.2021.755610, PMID: 34691131 PMC8527015

[B99] Teixeira M. A. Wei L. Kaloshian I. (2016). Root-knot nematodes induce pattern-triggered immunity in Arabidopsis thaliana roots. New Phytol. 211, 276–287. doi: 10.1111/nph.13893, PMID: 26892116

[B100] Trinh D. C. Lavenus J. Goh T. Boutté Y. Drogue Q. Vaissayre V.. (2019). PUCHI regulates very long chain fatty acid biosynthesis during lateral root and callus formation. Proc. Natl. Acad. Sci. U.S.A. 116, 14325–14330. doi: 10.1073/pnas.1906300116, PMID: 31235573 PMC6628829

[B101] Tsuda K. Mine A. Bethke G. Igarashi D. Botanga C. J. Tsuda Y.. (2013). Dual regulation of gene expression mediated by extended MAPK activation and salicylic acid contributes to robust innate immunity in Arabidopsis thaliana. PloS Genet. 9, e1004015. doi: 10.1371/journal.pgen.1004015, PMID: 24348271 PMC3861249

[B102] Uemura Y. Kimura S. Ohta T. Suzuki T. Mase K. Kato H.. (2023). A very long chain fatty acid responsive transcription factor, MYB93, regulates lateral root development in Arabidopsis. Plant J. 115, 1408–1427. doi: 10.1111/tpj.16330, PMID: 37247130

[B103] Vieira P. Kyndt T. Gheysen G. de Almeida Engler J. (2013). An insight into critical endocycle genes for plant-parasitic nematode feeding sites establishment. Plant Signal Behav. 8, e24223. doi: 10.4161/psb.24223, PMID: 23518580 PMC3907419

[B104] Vlieghe K. Boudolf V. Beemster G. T. Maes S. Magyar Z. Atanassova A.. (2005). The DP-E2F-like gene DEL1 controls the endocycle in Arabidopsis thaliana. Curr. Biol. 11;15, 59–63. doi: 10.1016/j.cub.2004.12.038, PMID: 15649366

[B105] Wang X. Meng H. Tang Y. Zhang Y. He Y. Zhou J.. (2022). Phosphorylation of an ethylene response factor by MPK3/MPK6 mediates negative feedback regulation of pathogen-induced ethylene biosynthesis in Arabidopsis. J. Genet. Genomics 49, 810–822. doi: 10.1016/j.jgg.2022.04.012, PMID: 35562093

[B106] Wang Y. Wang L. Zou Y. Chen L. Cai Z. Zhang S.. (2014). Soybean miR172c targets the repressive AP2 transcription factor NNC1 to activate ENOD40 expression and regulate nodule initiation. Plant Cell 26, 4782–4801. doi: 10.1105/tpc.114.131607, PMID: 25549672 PMC4311200

[B107] Wani S. H. Anand S. Singh B. Bohra A. Joshi R. (2021). WRKY transcription factors and plant defense responses: latest discoveries and future prospects. Plant Cell Rep. 40, 1071–1085. doi: 10.1007/s00299-021-02691-8, PMID: 33860345

[B108] Warmerdam S. Sterken M. G. Van Schaik C. Oortwijn M. E. P. Lozano-Torres J. L. Bakker J.. (2019). Mediator of tolerance to abiotic stress ERF6 regulates susceptibility of Arabidopsis to Meloidogyne incognita. Mol. Plant Pathol. 20, 137–152. doi: 10.1111/mpp.12745, PMID: 30160354 PMC6430479

[B109] Wieczorek K. (2015). “Chapter three- cell wall alterations in nematode-infected roots,” in Botanical research, vol. 73, 2015 . Eds. Escobar C. Fenoll. C. (London: Academic Press), 61–90. doi: 10.1016/bs.abr.2014.12.002

[B110] Willig J. J. Guarneri N. van Loon T. Wahyuni S. Astudillo-Estévez I. E. Xu L.. (2024). Transcription factor WOX11 modulates tolerance to cyst nematodes via adventitious lateral root formation. Plant Physiol. 8, kiae053. doi: 10.1093/plphys/kiae053, PMID: 38330218 PMC11060680

[B111] Wu Y. Li X. Zhang J. Zhao H. Tan S. Xu W.. (2022). ERF subfamily transcription factors and their function in plant responses to abiotic stresses. Front. Plant Sci. 13. doi: 10.3389/fpls.2022.1042084, PMID: 36531407 PMC9748296

[B112] Xiao K. Chen W. Chen X. Zhu X. Guan P. Hu J. (2020). CCS52 and DEL1 function in root-knot nematode giant cell development in Xinjiang wild myrobalan plum (Prunus sogdiana Vassilcz). Protoplasma 257, 1333–1344. doi: 10.1007/s00709-020-01505-0, PMID: 32367262

[B113] Yamaguchi Y. L. Suzuki R. Cabrera J. Nakagami S. Sagara T. Ejima C.. (2017). Root-knot and cyst nematodes activate procambium-associated genes in arabidopsis roots. Front. Plant Sci. 13;8. doi: 10.3389/fpls.2017.01195, PMID: 28747918 PMC5506325

[B114] Yan Z. Hossain M. S. Wang J. Valdes-Lopez O. Liang Y. Libault M.. (2013). MiR172 regulates soybean nodulation. Mol. Plant–Microbe Interact. 26, 1371–1377. doi: 10.1094/MPMI-04-13-0111-R, PMID: 23980625

[B115] Zhou W. Lozano-Torres J. L. Blilou I. Zhang X. Zhai Q. Smant G.. (2019). A jasmonate signaling network activates root stem cells and promotes regeneration. Cell 177, 942–956.e14. doi: 10.1016/j.cell.2019.03.006, PMID: 30955889

[B116] Zhu Q. H. Helliwell C. A. (2010). Regulation of flowering time and floral patterning by miR172. J. Exp. Bot. 62, 487–495. doi: 10.1093/jxb/erq295, PMID: 20952628

