# Correction: Functional studies of plant transcription factors and their relevance in the plant root-knot nematode interaction

**DOI:** 10.3389/fpls.2025.1673746

**Published:** 2025-08-12

**Authors:** Jose Domínguez-Figueroa, Almudena Gómez-Rojas, Carolina Escobar

**Affiliations:** ^1^ Facultad de Ciencias Ambientales y Bioquímica, Universidad de Castilla-La Mancha, Toledo, Spain; ^2^ Centro de Biotecnologia y Genomica de Plantas (CBGP), Universidad Politecnica de Madrid and Instituto de Investigacion y Tecnologia Agraria y Alimentaria-Consejo Superior de investigaciones Cientificas (UPM-INIA/CSIC), Madrid, Spain

**Keywords:** plant-RKNs interaction, galls, giant cells, transcription factors, new organogenesis, plant defense, plant-development

An incorrect **Funding** statement was provided. The funder “Castilla-La Mancha” was incorrect. The correct funder is “Castilla-La Mancha Government and EU FEDER” and the correct **Funding** sentence reads “by the Castilla-La Mancha Government and EU FEDER (SBPLY/17/180501/000287; SBPLY/21/180501/000033), to CE”.

The original version of this article has been updated.

